# Gou Qi Zi inhibits proliferation and induces apoptosis through the PI3K/AKT1 signaling pathway in non-small cell lung cancer

**DOI:** 10.3389/fonc.2022.1034750

**Published:** 2022-12-14

**Authors:** Lingling Zhang, Yanju Gong, Lei Zhang, Bing Liang, Huan Xu, Wangming Hu, Zhong Jin, Xiao Wu, Xiongbin Chen, Min Li, Liangqin Shi, Yaping Shi, Mingjian Li, Yong Huang, Yong Wang, Lan Yang

**Affiliations:** ^1^ Basic Medicine College, Chengdu University of Traditional Chinese Medicine, Chengdu, China; ^2^ School of Medicine, Jianghan University, Wuhan, China; ^3^ Innovative Institute of Chinese Medicine and Pharmacy, Chengdu University of Traditional Chinese Medicine, Chengdu, China

**Keywords:** traditional Chinese medicine (TCM), Gou Qi Zi (*Lycium barbarum*), non-small cell lung cancer (NSCLC), Network pharmacology, Molecular docking, Protein kinase B (AKT1)

## Abstract

**Background:**

Gou Qi Zi (*Lycium barbarum*) is a traditional herbal medicine with antioxidative effects. Although Gou Qi Zi has been used to prevent premature aging and in the treatment of non-small cell lung cancer (NSCLC), its mechanism of action in NSCLC remains unclear. The present study utilized network pharmacology to assess the potential mechanism of action of Gou Qi Zi in the treatment of NSCLC.

**Methods:**

The TCMSP, TCMID, SwissTargetPrediction, DrugBank, DisGeNET, GeneCards, OMIM and TTD databases were searched for the active components of Gou Qi Zi and their potential therapeutic targets in NSCLC. Protein-protein interaction networks were identified and the interactions of target proteins were analyzed. Involved pathways were determined by GO enrichment and KEGG pathway analyses using the Metascape database, and molecular docking technology was used to study the interactions between active compounds and potential targets. These results were verified by cell counting kit-8 assays, BrdU labeling, flow cytometry, immunohistochemistry, western blotting, and qRT-PCR.

**Results:**

Database searches identified 33 active components in Gou Qi Zi, 199 predicted biological targets and 113 NSCLC-related targets. A network of targets of traditional Chinese medicine compounds and potential targets of Gou Qi Zi in NSCLC was constructed. GO enrichment analysis showed that Gou Qi Zi targeting of NSCLC was mainly due to the effect of its associated lipopolysaccharide. KEGG pathway analysis showed that Gou Qi Zi acted mainly through the PI3K/AKT1 signaling pathway in the treatment of NSCLC. Molecular docking experiments showed that the bioactive compounds of Gou Qi Zi could bind to AKT1, C-MYC and TP53. These results were verified by experimental assays.

**Conclusion:**

Gou Qi Zi induces apoptosis and inhibits proliferation of NSCLC *in vitro* and *in vivo* by inhibiting the PI3K/AKT1 signaling pathway.

## Introduction

Lung cancer is the leading cause of cancer-related death worldwide. More than 85% of these tumors are classified as non-small cell lung cancers (NSCLCs) (1, 2). Subtypes of NSCLC include adenocarcinoma (ADC), squamous cell carcinoma (SCC) and large cell carcinoma (LCC), as well as other less common subtypes (3). Although standardized treatment of NSCLC, including chemotherapy and radiotherapy, has shown clinical benefits (4), treatment is also associated with unwanted side effects, including mood disorders, chronic pain and decreased quality of life (5). New drugs with high efficacy and low toxicity are therefore urgently needed for the treatment of NSCLC.

Traditional Chinese Medicine (TCM) has been used in patient treatment for thousands of years. The active components of TCM have multi-targeted therapeutic effects in patients with various lung diseases (6) including chronic obstructive pulmonary disease (COPD) (7), acute asthma (8) and NSCLC (9). Gou Qi Zi (GQZ; *Lycium barbarum*) is a common Chinese herb that has been used as a functional dietary supplement in health recipes worldwide. Traditionally it is regarded as an agent that nourishes the liver and moistens the lungs and eyes. GQZ was shown to have nutritional, preventive and therapeutic properties, including in immune regulation, antioxidation, anti-aging, cryoprotection, hypnotic protection and cancer prevention (10, 11). Extracts of *Lycium barbarum* (LB) have shown antioxidant activity, suggesting that LB may be a potential protective and therapeutic agent for lung injury (12, 13). The components of LB can treat a variety of lung related diseases. For example, lycium barbarum polysaccharide (LBP) can alleviate the symptoms of dyspnea in patients with stable COPD by reducing hypoxia-inducible factor 1- α (HIF-1α) (14), and can improve symptoms of allergic asthma by reducing lung lesions, improving airway inflammation and regulating intestinal flora (15). In addition, the combination of LBP and Lak/IL-2 has been found to prolong the life span and improve the quality of life of patients with lung cancer (16). Although several studies have reported that GQZ can prevent lung injury, the mechanism of action of GQZ is not completely clear. Studies are therefore needed to evaluate the mechanism of action of GQZ in the treatment of NSCLC.

Network pharmacology, consisting of the combination of systems biology and pharmacology, can comprehensively evaluate the mechanism of action of multi-component, multi-target and multi-channel Chinese herbal medicines (17, 18). A “network target, multi-component” model can systematically clarify the molecular mechanisms of action of TCM in the treatment of various diseases. To date, studies have evaluated the mechanisms of cinobufagin in the treatment of hepatocellular carcinoma, zanthoxylum bungeanum in the treatment of pain, and Xiaochaihu Decoction in the treatment of acute pancreatitis (19–21). The present study used network pharmacology methods to evaluate the pharmacological and molecular mechanisms of action of GQZ in the treatment of NSCLC.

## Materials and methods

### Data preparation

#### Data acquisition and ADME screening

The molecular structures, molecular formulas, and targets of action of the compounds of GQZ were obtained from the Traditional Chinese Medicine Information Database (TCMID) (http://www.megabionet.org/tcmid/) (22). Potential pharmacologically active compounds in GQZ were identified based on the ADME-related properties of compounds retrieved from the Traditional Chinese Medicine System Pharmacology (TCMSP) database (http://tcmspw.com/tcmsp.php) and the SwissADME database (http://www.swissadme.ch/) (23). OB values ≥30% were regarded as having good absorption after oral administration, whereas a computed value of DL not less than 0.18 indicated that the compound was chemically suitable for drug development (24). The screening criteria used in the SwissADME database were: gastrointestinal absorption: High; Lipinski: Yes; Ghose: Yes; Veber: Yes; Egan: Yes; and Muegge: Yes. In addition, the PubChem ID numbers, 2D structures, IUPAC International Chemical Identifier (InChI), and canonical structures of the compounds were obtained from the PubChem database (https://pubchem.ncbi.nlm.nih.gov/) (25).

#### Identification of GQZ compound-related targets and NSCLC-related targets

Biological targets of the GQZ compounds were identified by searches of three public databases, TCMSP, SwissTargetPrediction, and TCMID, as determined by chemical similarities and pharmacophore models (26, 27). In the SwissTargetPrediction database, the probabilities derived from cross-validation analyses were used to rank the targets and estimate the accuracy of the predictions, with targets having probabilities ≥0.5 selected (26). Genes associated with the GQZ compounds were selected if they had confidence scores of >0.7 (27). The standard gene names and UniProt ID of target proteins were obtained from the UniProt KB database (https://www.uniprot.org/) by limiting the species to *Homo Sapiens*.

Information on NSCLC-associated target genes was also collected from five database resources, DrugBank, DisGeNET, GeneCards, OMIM and TTD (28–32). The search results of the databases were combined and duplicate targets were deleted, yielding a list of all targets of NSCLC. The intersection of the targeted prediction results of the active components of GQZ and the retrieval results of the relevant targets of NSCLC was determined, with common targets regarded as potential therapeutic targets of GQZ in NSCLC. Active ingredients of GQZ and their targets in NSCLC were identified using Venny version 2.1 software (http://bioinfogp.cnb.csic.es/tools/venny/index.html) and a Venn diagram was drawn. The NSCLC-associated targets and the predicted GQZ targets were verified using UniProtKB ID, with the protein and gene names obtained from the UniProt database (http://www.uniprot.org/). A gene library of the anti-NSCLC targets of GQZ was established by comparing and analyzing the genes common to NSCLC-associated targets and predicted GQZ targets.

#### Protein-protein interaction data

A PPI network was constructed from the GQZ target and NSCLC-related gene set using the Search Tool for the Retrieval of Interacting Genes (STRING, http://string-db.org/) database (33). Screening condition was limited to *Homo Sapiens*, and the free point was hidden. PPIs in the STRING database with minimum interaction scores of >0.7, >0.4 and >0.15 were defined as high, medium, low, respectively. In this study, the PPI network had a confidence level of 0.4 (medium).

#### Network construction and analysis

To determine the therapeutic characteristics of multiple compounds of GQZ, a network was constructed using Cytoscape (version 3.7.1) (34). The GQZ (active compound) predicted target network was constructed based on each active compound and its potential targets; the target network of GQZ for treating NSCLC was constructed based on NSCLC related and GQZ predicted targets common to both; and the PPI results were exported as tabular text output (.tsv), into Cystocape (version 3.7.1). Target protein interactions were analyzed, key subnets were screened, and complex network parameters were calculated using the network analyzer. Highly connected subnetworks were identified using the Cytohubba plugin of Cytoscape, and the 14 leading gene targets were selected.

#### GO and KEGG pathway enrichment analyses

The biological functions of potential targets of NSCLC have been determined by using the Metascape database (http://metascape.org/) (35) to perform Gene Ontology (GO) functional annotation analysis of biological processes, molecular functions and cellular components and Kyoto Encyclopedia of Genes and Genomes (KEGG) pathway enrichment analysis (36). In our present study, the genes common to GQZ treatment and NSCLC targets were imported into the Metascape platform, and GO and KEGG pathway enrichment analyses were performed for *Homo sapiens* to determine the mechanism of action of GQZ. The adjusted *p* values of the identified terms were sorted using the error discovery rate (FDR) algorithm, and a bubble chart was generated with RStudio software. Based on the number of targets involved in each channel, the target channel network was constructed and analyzed using Cytoscape (version 3.7.1) software.

#### Molecular docking technology

The binding affinity, binding site and interaction of each active compound of GQZ with its predicted target were analyzed using PyMOL 1.7.2.1, AutoDockTools 1.5.6 and the classical molecular dynamics in Discovery studio-2020. The 3D structure of each protein was downloaded in the RCSB PDB database (https://www.rcsb.org/), with each 3D structure calculated and exported using ChemBio 3D software while minimizing energy. Receptor proteins were dehydrated using PyMOL 2.4.0 software, and proteins hydrogenated and their charges calculated using AutoDock software. The parameters of the receptor protein docking site were set to include the active pocket sites to which small molecule ligands bind. Receptor proteins were docked with the small molecule ligands of the active compounds of GQZ using Autodock vina.

### Experimental verification

#### Cell culture and reagents

Human NSCLC A549 cells were obtained from the American Type Culture Collection (Manassas, VA, USA) were cultured in RPMI 1640 medium (Gibco, USA) supplemented with 10% fetal bovine serum (FBS, Gibco, USA), streptomycin (100 μg/ml), and penicillin (100 U/ml), at 37°C in an incubator with 5% CO2. Crude extract powder of *Lycium barbarum* (LB, 20211126), synthesized as described and of purity ≥90% (**Figure S1**), was purchased from Yikangtang Pharmaceutical Co., Ltd (Chengdu, China), dissolved in ultrapure water and stores at -20°C; the stock solution was thawed and diluted with medium before each experiment. The main constituents of LB include LBP, scopoletin, and 2-O-β-D-glucopyranosyl-L-ascorbic acid (AA-2βG) (37).

#### Cell counting kit-8 assay

The antitumor activities of LB on A549 cells were evaluated using CCK-8 assays. Briefly, cultured A549 cells were detached by incubation with 0.25% trypsin, centrifugated at 400 g for 3 min, suspended in medium and seeded in 96-well plates at a density of 8×10^3^ cells/well. After overnight incubation, the cells were treated with LB (80 μg/ml) for 24 h. A 10 μl of CCK8 solution was added to each well, followed by incubation for 2-3 h at 37°C in a humidified atmosphere containing 5% CO_2_. The optical density at each well 450 nm was measured by using a microplate reader (Thermo, USA).

#### Flow cytometry assay

The apoptosis of treated cells was evaluated using an Annexin V-FITC/PI apoptosis kits (Beyotime, China) according to the manufacturer’s protocol. Briefly, the cells were collected, washed twice with phosphate-buffered saline (PBS), and incubated in 500 μl binding buffer containing 5 μl Annexin-V FITC and 5 μl PI incubating in the dark for 5 min. Cell apoptosis was subsequently determined by flow cytometry (BD FACSVerse, USA).

#### Quantitative real-time polymerase chain reaction

Total RNA was extracted from A549 cells using TRIzol reagent (Invitrogen, USA) and reverse transcribed to complementary DNA (cDNA) using HiScript III RT SuperMix (Vazyme, China). Sequences corresponding to the designated genes were PCR amplified using ChamQ Universal SYBR qPCR Master Mix (Vazyme, China) and the primers listed in **Table S1**. The levels of designated mRNAs were normalized to that of GAPDH mRNA, with relative quantification determined using the 2^-ΔΔCT^ method.

#### BrdU labelling and staining

Aliquots containing 8×10^4^ cells were added to glass bottom culture dishes and treated with BrdU (Thermo, USA) for 12 h, followed by treatment with LB for 24 h. The numbers of BrdU labeled cells were determined by fluorescence microscopy (TCS SP8, Germany), and six independent fields were randomly selected by confocal microscopy to calculate the percentages of positively labeled cells.

#### Western blot analysis

A549 cells treated with LB on for 24 h and A549 xenograft tumor treated with LB for 16 days were lysed by incubation in RIPA lysis buffer supplemented with protease inhibitors (Beyotime, China), followed by centrifugation to remove cell debris. The concentrations of proteins in the supernatants were measured by BCA Protein Assay Kit (Absin, China). Equal aliquots of proteins were separated by sodium dodecylsulfate polyacrylamide gel electrophoresis (SDS-PAGE) and transferred to polyvinylidene fluoride (PVDF) membranes (Millipore, USA). The membranes were incubated with 5% non-fat milk for 1 h at room temperature, followed by incubation overnight with primary antibodies (**Table S2**). After rinsing, the membranes were incubated with horseradish peroxidase-conjugated secondary antibodies at room temperature for 2 h. Signals were visualized by enhanced chemiluminescence, with signal intensity analyzed using Image J software.

#### Xenograft mouse models

The protocols of all animal experiments were approved by the Experimental Animal Ethics Committee of Chengdu University of Traditional Chinese Medicine (ethics approval number: 2020-124). Six-to-eight week old male BALB/c nude mice were purchased from Beijing SiPeiFu Animal Co., Ltd. Mice were inoculated in the armpit with 100 μl of a suspension containing 5×10^6^ A549 cells in logarithmic growth phase. Approximately 3-5 days later, mice that exhibited induration with a diameter of 5 mm, suggesting successful tumor establishment, were randomly divided into two groups, with one group of mice injected intraperitoneally (ip) with LB (10 mg/kg/day) for 16 days. Animal weights and the long and short tumor diameters were measured every four days. All mice were subsequently sacrificed and tumor tissue was removed.

#### Immunohistochemistry

Paraffin-embedded tumor tissue samples were cut into 5-μm sections, deparaffinized and subjected to antigen recovery with citric acid buffer under high temperature for 10 min. Following blocking with goat serum at room temperature for 1 h, the samples were incubated overnight at 4°C with the primary antibody (1:200), followed by incubated with biotinylated secondary antibody for 1 h at room temperature and incubation with ABC solution for 30 min at room temperature. Target protein expression was visualized after addition of DAB solution.

#### Statistical analysis

The data were reported as the mean ± SEM, and compared in two groups by two-tailed unpaired Student’s-t test or one- or two-way ANOVA, followed by Bonferroni’s *post hoc* tests as appropriate. All statistical analyses were performed using GraphPad Prism software, with P < 0.05 considered statistically significant.

## Results

### Screening of active compounds and construction of compound-target network

**Figure 1:**
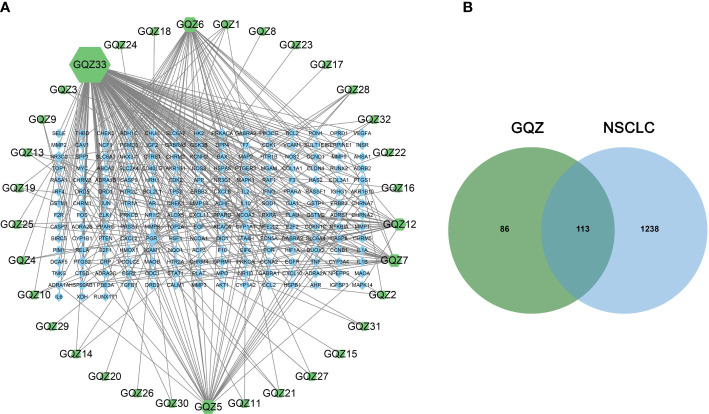
**(A)** Predicted target network formed by compounds in GQZ. Active compounds are represented by green nodes and predicted targets are represented by blue nodes. Each edge represents the interaction between each compound and target, with the node size being directly proportional to the degree of interaction. **(B)** Identification of the 113 matching predicted GQZ and NSCLC related targets.

Evaluation of the TCMSP and TCMID databases revealed 199 compounds in GQZ, with ADME screening identifying 33 active compounds in GQZ (**Table S3**). Using these 33 compounds, 199 targets were predicted by target search based on chemical similarity. A composite target network composed of 232 nodes and 329 edges was constructed (**Table S4**; **Figure 1A**). Network analysis showed that quercetin (GQZ33, degree=152), beta-sitosterol (GQZ6, degree=37), atropine (GQZ7, degree=25) and glycitein (GQZ12, degree=22) were most closely connected with different targets.

**Figure 2:**
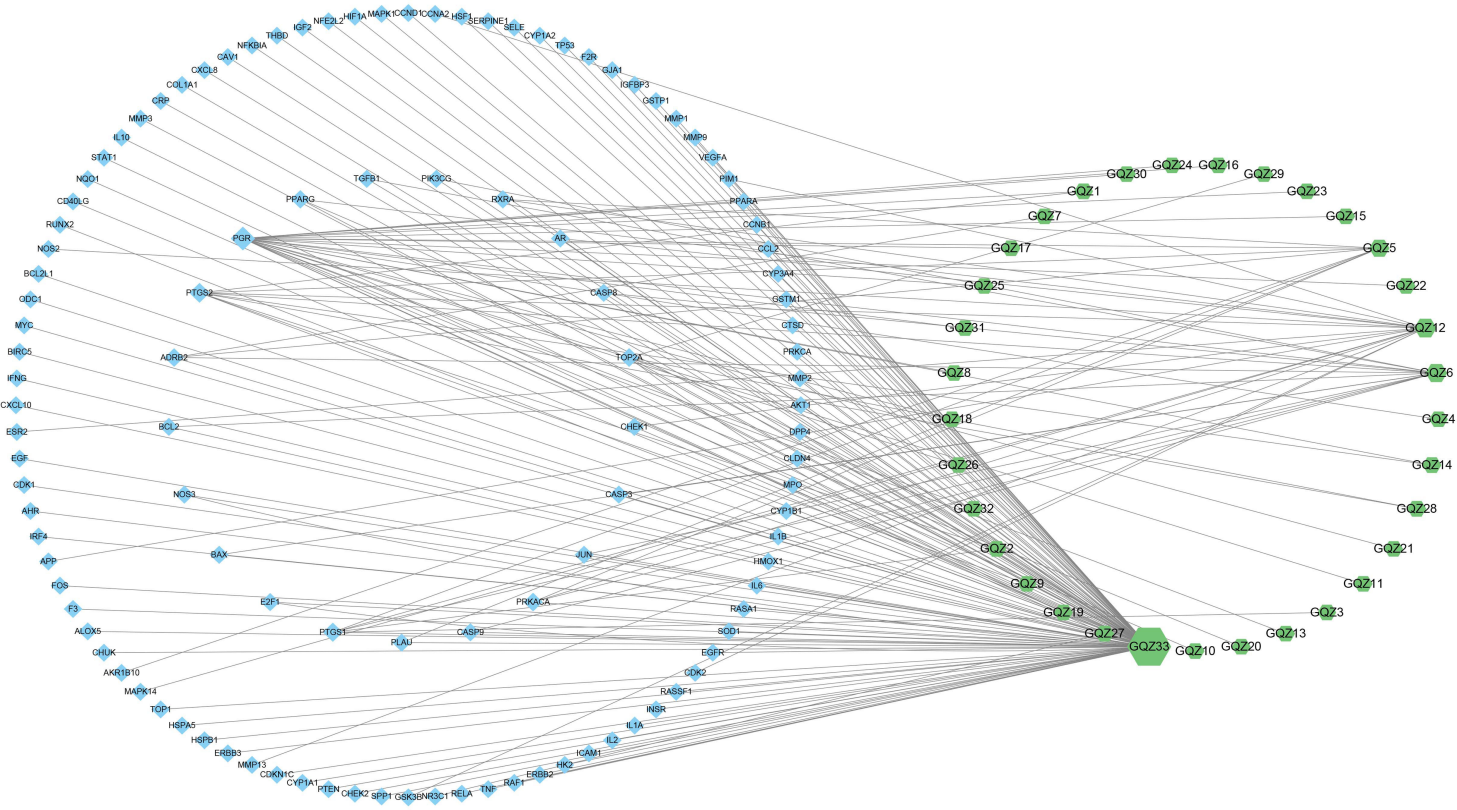
Complex target network formed by active compounds in GQZ and targets of these compounds in the treatment of NSCLC. Green nodes represent active compounds in GQZ, while blue nodes represent anti-NSCLC targets of these active compounds. The size of nodes is directly proportional to the degree of interaction.

Integration of NSCLC-related targets in the Drugbank, Genecards, DisGeNet, OMIM and TTD databases resulted in the retrieval of 1351 targets. A comparison of these targets with the predicted GQZ targets resulted in the identification of 113 common targets, identified as key targets of GQZ compounds in the treatment of NSCLC (**Figure 1B**). The relationships between the target proteins of NSCLC and GQZ target proteins were assessed by constructing a network containing 146 nodes and 177 edges. (**Figure 2**; **Table S5**). Quercetin (GQZ33, degree=106), beta-sitosterol (GQZ6, degree=13) and glycitein (GQZ12, degree=16) were found to have the highest number of connections to most targets, indicating that these three compounds are likely the most critical ingredients of GQZ. In addition, some target proteins were affected by several compounds. For example, progesterone receptor (PGR, degree=25) and prostaglandin G/H synthase 2 (PTGS2, degree=9) were modulated by at least nine compounds each, including sitosterol alpha1, stigmasterol, beta-sitosterol and 14b-pregnane and 7-O-methylluteolin-6-C-beta-glucoside_qt.

### PPI network of the anti-NSCLC targets of GQZ

**Figure 3:**
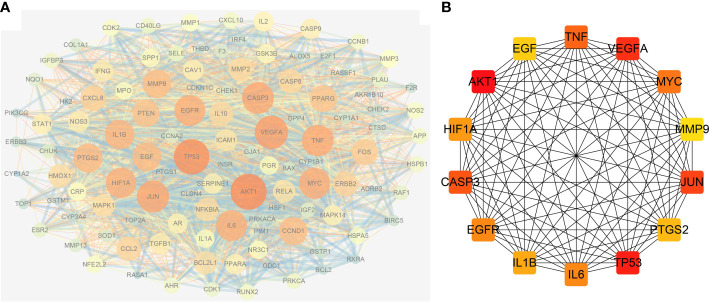
Reconstruction of the PPI network. **(A)** Construction of the PPI network using Cytoscape and analysis of the network using Network Analyzer. **(B)** Clusters retrieved from **(A)**; the color depth of nodes is proportional to the degree of interaction.

The possible mechanism of action of GQZ in the treatment of NSCLC was explored by importing 113 anti-NSCLC gene symbols into the STRING database and constructing a PPI network (**Figure S2**). The original network, consisting of the PPI network of the anti-NSCLC targets of GQZ, obtained from the STRING database was complex. Therefore, a second network was constructed from the tsv file of PPI data from the STRING database generated using Cytoscape (version 3.7.1) for better visualization and understanding. The reconstructed PPI network consisted of 113 nodes and 2313 edges (**Figure 3A**; **Table S6**). Cytohubba was used to generate highly connected sub-networks for further cluster analysis, with the attributed values of the clusters shown in **Table 1**. The cluster consisted of 14 nodes and 91 edges, including the first 14 genes screened by degree (**Figure 3B**). In the PPI network, AKT1 and TP53 were found to be the centers of interaction with other targets of GQZ in the cluster, indicating the importance of AKT1 and TP53 in connecting other target nodes. Quercetin has been shown to regulate the post-translational modification of p53 in the treatment of lung cancer (38). AKT1 can be activated by various stimulating factors, including growth factors and cytokines, as well as being an important gene related to NSCLC.

**Table 1:** The network parameters.

Network Parameters	Value
Number of nodes	14
Number of edges	91
Clustering coefficient	1
Network diameter	1
Network radius	1
Network centralization	0
Network density	1
Characteristic path length	1
Avg. number of neighbors	13

### GO enrichment analysis

**Figure 4:**
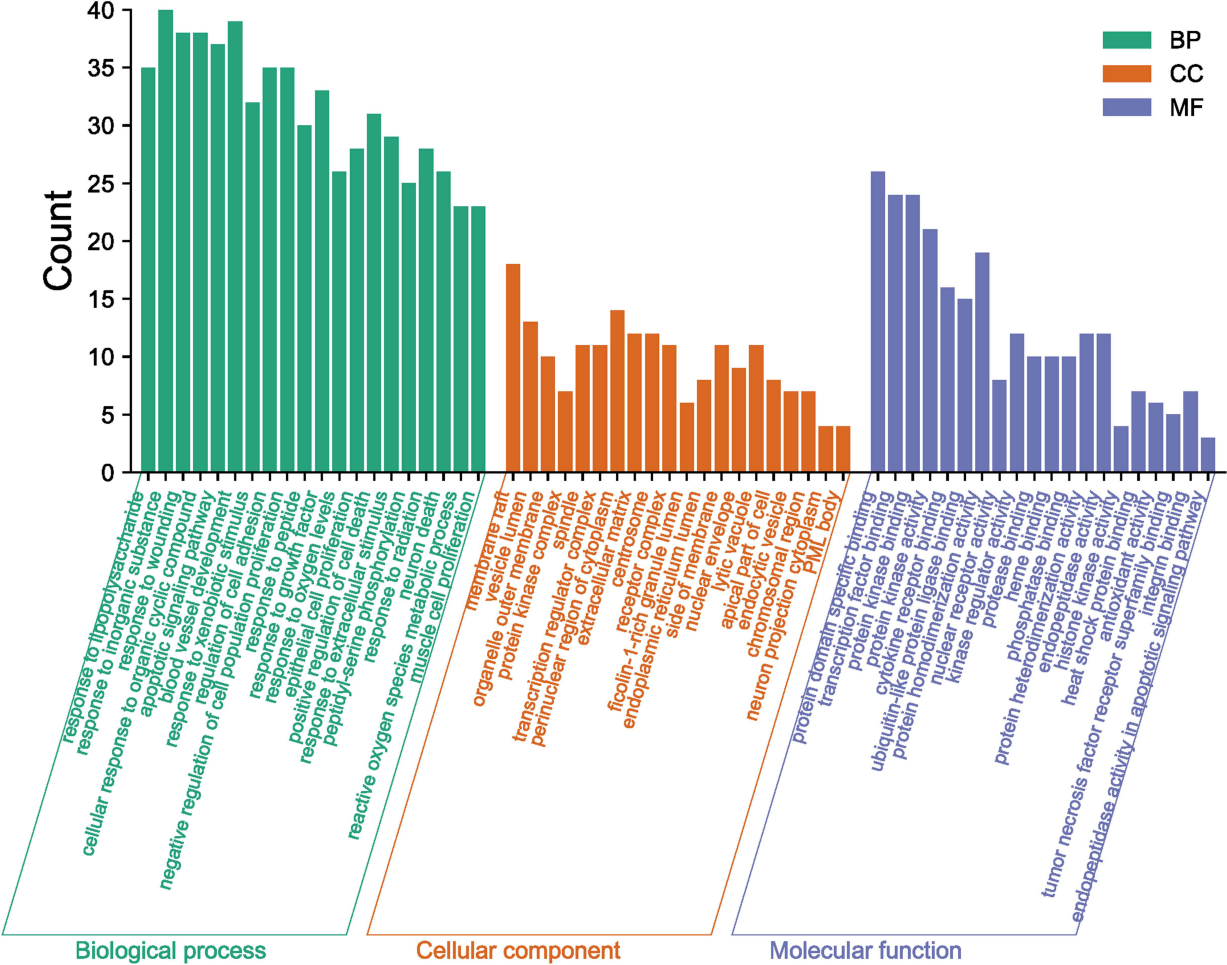
GO Enrichment analysis of GQZ in the treatment of NSCLC, including cellular components (CC), biological processes (BP), and molecular function (MF).

The mechanisms of action of GQZ in the treatment of NSCLC were evaluated by GO enrichment analysis of biological processes, molecular functions, and cellular components associated with the 113 predicted targets (**Tables S7–S9**). The 20 most enriched biological process items, molecular function items and cell component items were determined (**Figure 4**). The first five biological processes terms were (1): response to lipopolysaccharide (2), response to inorganic substances (3), response to wounding (4), cellular response to organic cyclic compounds, and (5) apoptotic signaling pathway. The top five molecular function terms were (1): protein domain specific binding (2), transcription factor binding (3), protein kinase binding (4), protein kinase activity, and (5) cytokine receptor binding. The top five cellular component terms were (1): membrane rafts (2), vesicle lumen (3), organelle outer membranes (4), spindles, and (5) protein kinase complex. These targets were found to be closely associated with the regulation of flavonoid activity and with enhancement of the activity and binding of flavonoids receptors.

### KEGG enrichment analysis

**Figure 5:**
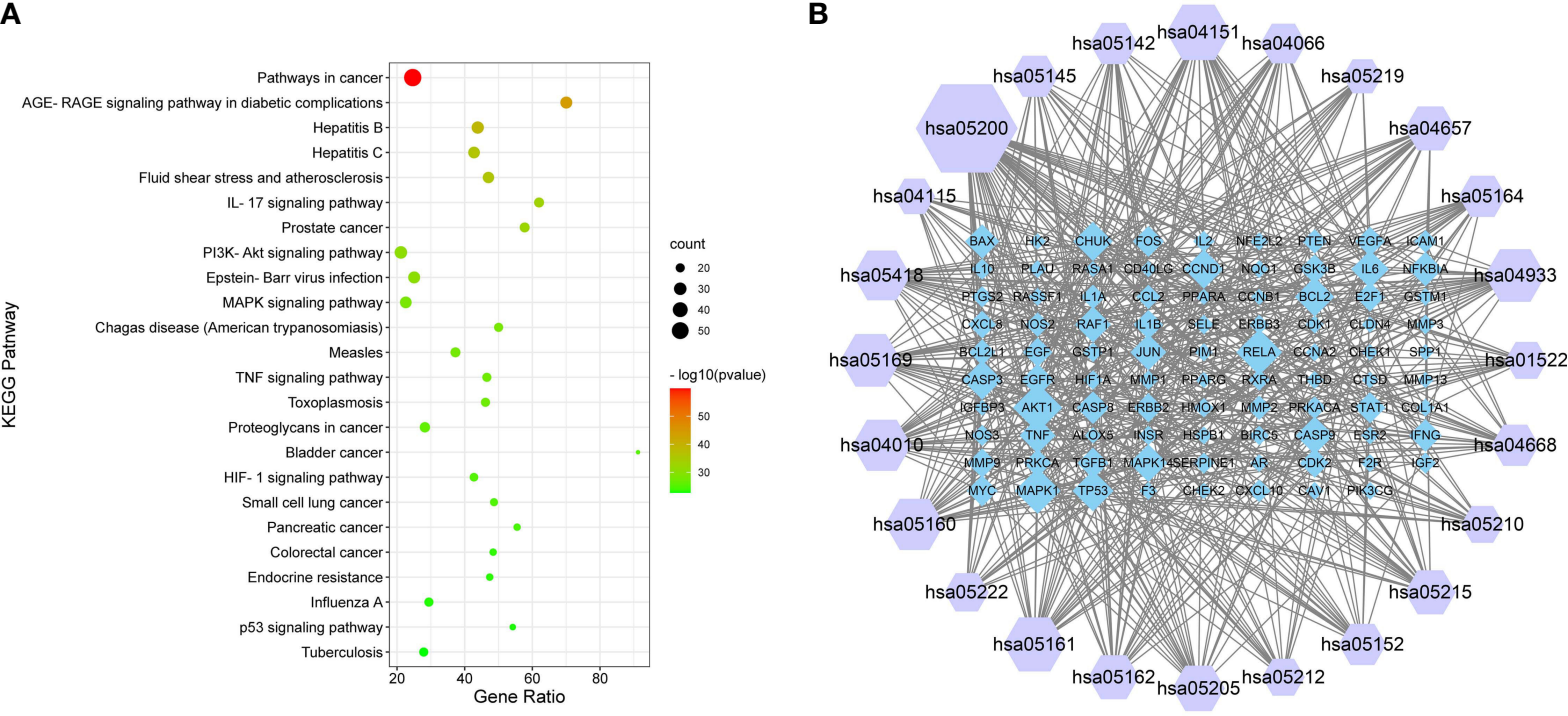
KEGG pathway enrichment analysis of GQZ in the treatment of NSCLC. **(A)** Pathway enrichment results at p<0.01. **(B)** Targeted pathway networks associated with the mechanism of action of GQZ in the treatment of NSCLC. Purple nodes represent paths, while blue nodes represent targets participating in these paths. Each edge represents the interaction between the target and the path, with node size being directly proportional to the degree of interaction.

KEGG enrichment analysis showed 113 GQZ targets were enriched at significance levels of p<0.01 (**Figure 5A**). The nodes of the KEGG pathway are shown in **Table S10**. The first five significantly enriched KEEG pathways were (1): pathways in cancer (hsa05200) (2), the AGE-RAGE signaling pathway in diabetic complications (hsa04933) (3), hepatitis B (hsa05161) (4), hepatitis C (hsa05160), and (5) fluid shear stress and atherosclerosis (hsa05418). Based on the number of targets contained within each pathway, a network diagram of the target pathway was constructed and analyzed by using Cytoscape (version 3.7.1) software. The target-pathway network was found to consist of 113 nodes and 564 edges (**Figure 5B**; **Table S11**). Most targets were associated with cancer, the PI3K-AKT signaling pathway and hepatitis B. The targets with the largest number of participating pathways were AKT1, RELA, and MAPK1, which participate in 21, 19, and 19 pathways, respectively. Based on these findings and the results of GO analysis, the mechanism of action of GQZ in the treatment of lung cancer, especially NSCLC, involves the PI3K/AKT1 signaling pathway.

### Molecular docking

**Figure 6:**
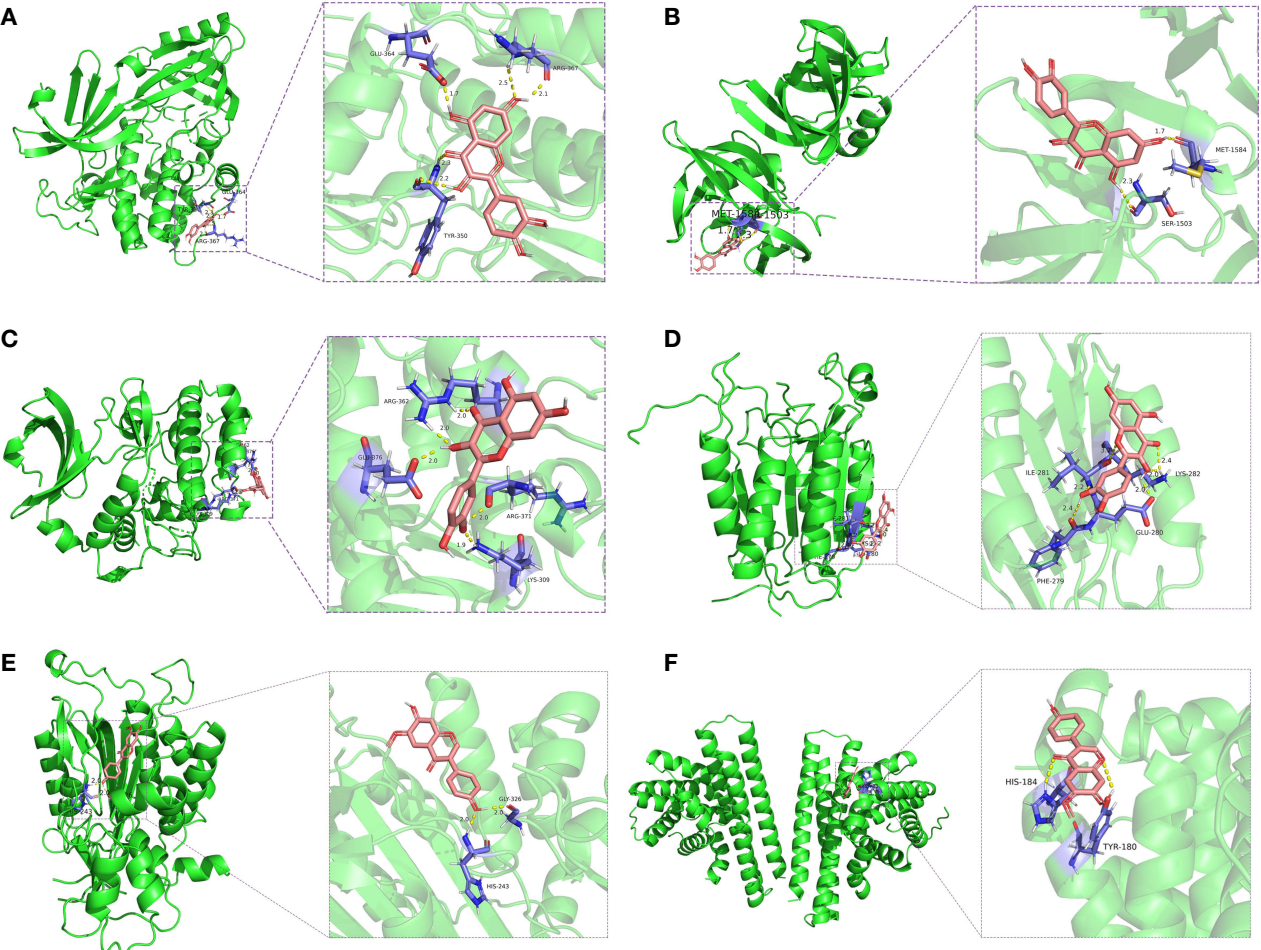
Three-dimensional schematic diagram of the molecular docking model, showing active sites and binding distances. **(A–D)** Binding mode of quercetin (GQZ33) to **(A)** AKT1, **(B)** TP53, **(C)** MYC, and **(D)** Caspase8. **(E, F)** Binding mode of glycitein (GQZ12) to **(E)** Caspase9 and **(F)** BCL2.

Enrichment analysis of GO and KEGG pathways showed that the anti-NSCLC effect of GQZ may be closely related to functional death such as apoptosis. The mechanism of action of GQZ compounds in the treatment of NSCLC was further evaluated by assessing the interactions of active compounds with good pharmacokinetic characteristics and their potential targets. Three NSCLC related targets, AKT1, TP53 and MYC, and three targets involved in apoptosis regulation in the PI3K/AKT signaling pathway, including BCL2, Caspase8 and Caspase9, were selected. Six molecules were selected for molecular docking with the main compounds of GQZ, with AutoDockTools-1.5.7 used to evaluate the interactions of these six targets with their corresponding active compounds. Compound-target interactions with higher free binding energy scores and their binding modes were also measured using PyMOL-1.7.2.1. The GQZ compounds in this network showed strong affinity with their predicted targets. For example, GQZ33 showed strong associations with the active pockets of AKT1, TP53 and MYC, with docking scores of -6.5, -7.3, and -8.2 kcal/mol, respectively, forming five, two and five hydrogen bonds, respectively, with different amino acid residues (**Figures 6A** **–C**). GQZ33 and GQZ12 were also found to bind with Caspase8, BCL2 and Caspase9, with docking scores of -5.2, -6.9, and -7.5 kcal/mol, respectively (**Figures 6D** **–F**). These findings showed that the molecular docking results were consistent with the screening results of network pharmacology, with the reliability of the latter confirmed by molecular docking.

### LB inhibits the proliferation and induces apoptosis of A549 cells through the PI3K/AKT1 pathway *in vitro*

**Figure 7:**
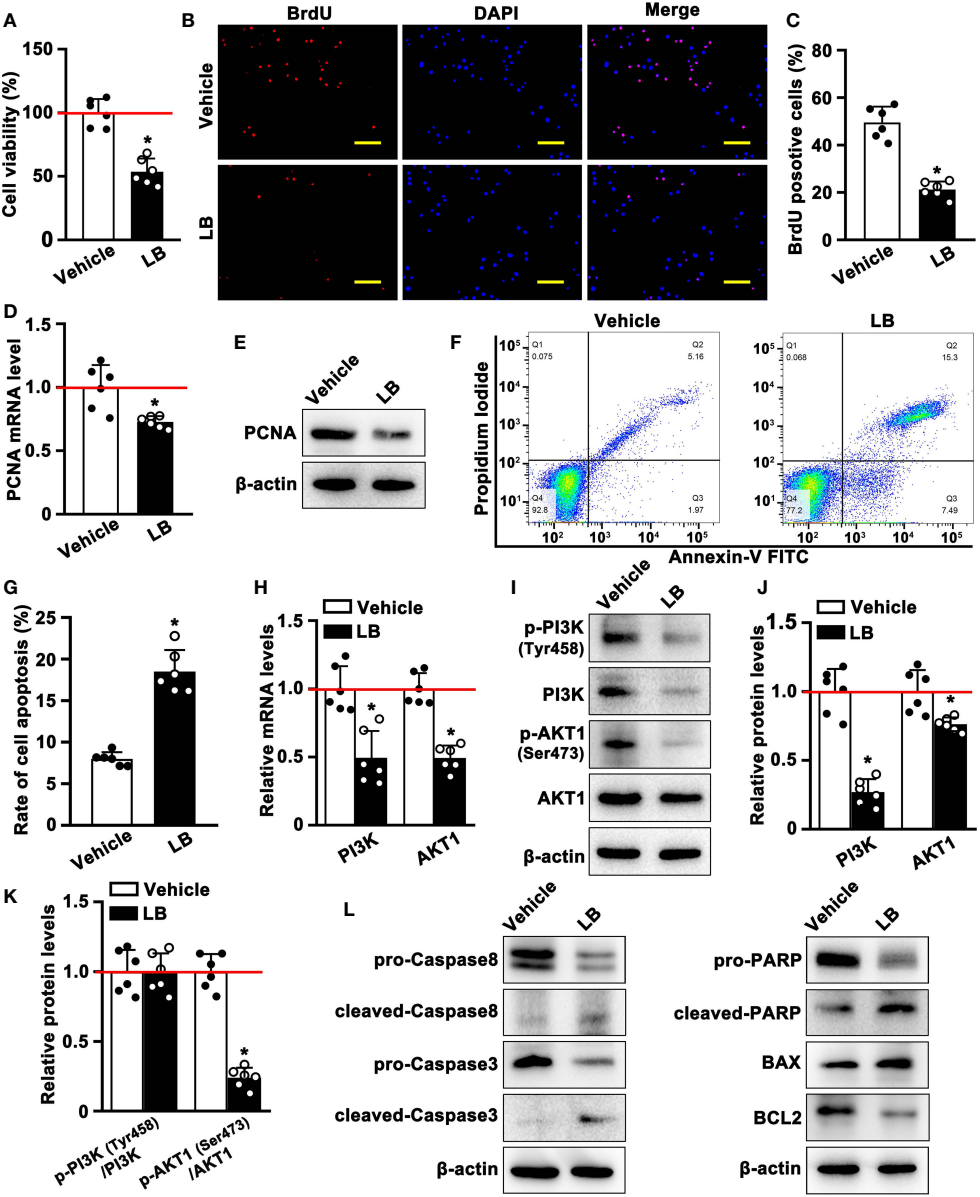
LB inhibits the proliferation and induces the apoptosis of A549 cells through the PI3K/AKT1 pathway *in vitro*. **(A)** Effects of LB on cell viability. A549 cells were seeded in 96-well plates and treated with LB (80 μg/ml) or vehicle for 24 h (n=6 each), and cell viability was assessed by CCK8 assays. **(B, C)** Effects of LB on cell proliferation. A549 cells were incubated with BrdU (0.96 mM) for 8 h, followed by treatment with LB (80 μg/ml) or vehicle for 24 h (n=6 each). The bar represents 100 μm. **(D)** Effects of LB on PCNA expression. A549 cells were treated for 24 h with LB (80 μg/ml) or vehicle (n=6 each). Relative PCNA mRNA expression was analyzed by qRT-PCR, with GAPDH mRNA serving as an internal reference, **(E)** and PCNA protein expression was analyzed by western blotting. **(F, G)** Effects of LB on A549 cell apoptosis. A549 cells were treated with LB (80 μg/ml) or vehicle for 24 h (n=6 each), followed by double staining with Annexin V-FITC and PI and detection of apoptotic cells by flow cytometry. **(H)** Effects of LB on PI3K and AKT1 mRNA expression. A549 cells were treated with LB (80 μg/ml) or vehicle for 24 h (n=6 each), and relative PI3K and AKT1 mRNA expression levels were analyzed by qRT-PCR, with GAPDH mRNA serving as an internal reference. **(I–K)** Effects of LB on the expression of p-PI3K (Tyr458), PI3K, p-AKT1 (Ser473) and AKT1. A549 cells were treated with LB (80 μg/ml) or vehicle (n=6), and protein expression was analyzed by western blotting. **(L)** Effects of LB on the expression of Caspase8, Caspase3, PARP, BCL2, and BAX. A549 cells were exposed to LB (80 μg/ml) for 24 h, followed by western blotting analysis of protein expression, with β-actin serving as the protein loading control. Data are expressed as mean ± SEM. *P< 0.05.

To verify the above network pharmacology and molecular docking results, A549 cells were incubated with LB, a crude extract of GQZ, to determine whether LB inhibits A549 cell proliferation *in vitro*. CCK8 assays showed that the viability of A549 cells was significantly inhibited by incubation with LB for 24 h (**Figure 7A**), with cellular immunofluorescence assays showing that, compared with vehicle, treatment with LB significantly reduced the number of BrdU-positive cells (**Figures 7B, C**). LB also significantly reduced the levels of the expression of PCNA mRNA and protein (**Figures 7D, E**). Taken together, these results indicated that LB inhibited the proliferation of A549 cells. Further evaluation by flow cytometry showed that LB significantly increased the apoptosis rate of A549 cells (**Figures 7F, G**). Moreover qRT-PCR and western blotting showed that the levels of PI3K and AKT1 mRNA and protein in A549 cells were reduced after treatment with LB, accompanied by reduced levels of expression of p-PI3K and p-AKT1 proteins (**Figures 7H** **–J**). LB significantly reduced the p-AKT1/AKT1 ratio, but did not significantly alter the p-PI3K/PI3K ratio, compared with cells incubated with vehicle (**Figure 7K**). Compared with vehicle, incubation with LB enhanced the expression of cleaved-Caspase8, cleaved-Caspase3, cleaved-PARP, Bim and BAX mRNA and protein, but decreased the expression of pro-Caspase8, pro-Caspase3, pro-PARP and BCL mRNA and protein (**Figures S4**, **7L**). These results indicated that LB could inhibit proliferation while inducing apoptosis in A549 cells through the PI3K/AKT1 signaling pathway.

### LB inhibited the proliferation and induced apoptosis in A549 xenograft tumor

**Figure 8:**
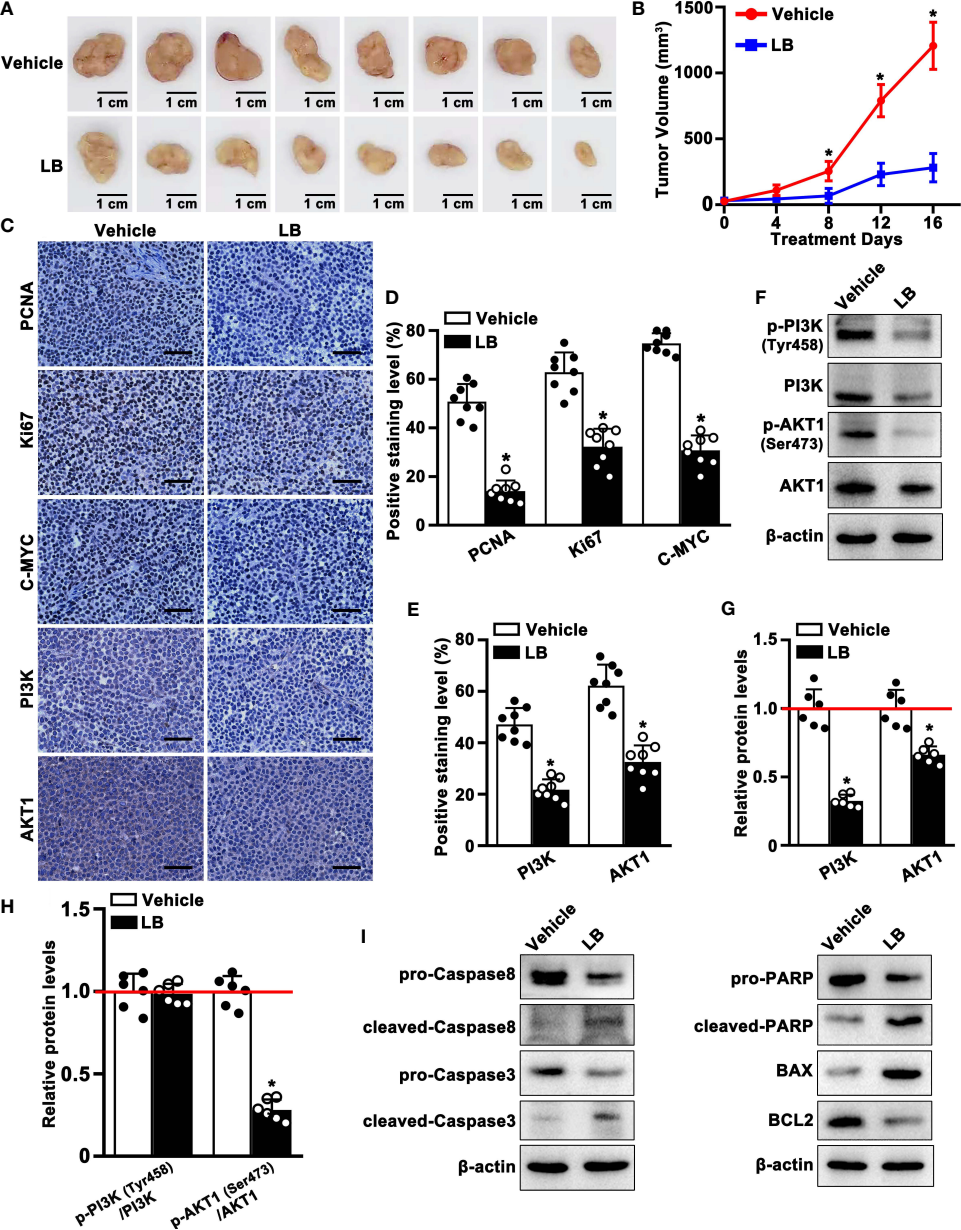
LB inhibited the proliferation and induced apoptosis of A549 xenograft tumors. BALB/c nude mice were injected subcutaneously with A549 cells, followed by injection of LB (10 mg/kg/day) or vehicle for 16 days. **(A)** Photographs showing xenograft tumors after injection of LB or vehicle for 16 days. **(B)** Tumor growth curve in vehicle group and LB group (n=8 each). **(C–E)** Immunohistochemical analyses of PCNA, Ki67, C-MYC, PI3K and AKT1 protein expression in tumor tissues of mice treated with LB and vehicle (n=8 each). The bar represents 50 μm. **(F–H)** Western blotting analyses of p-PI3K (Tyr458), PI3K, p-AKT1 (Ser473) and AKT1 protein expression levels in xenograft tumors of mice treated with LB (10 mg/kg/day) or vehicle (n=6 each). **(I)** Western blotting analyses of Caspase8, Caspase3, PARP, BCL2, and BAX protein expression levels in xenograft tumors of mice treated with LB or vehicle. Data are expressed as mean ± SEM. *P< 0.05.

The anti-tumor effect of LB *in vivo* was evaluated by inducing xenograft tumors in mice with A549 cells and treating these mice with LB. Tumor volumes and tumor weights were significantly lower in mice administered LB than vehicle, with relative reductions of 77% and 62%, respectively (**Figures 8A, B**; **S5A, B**), although mouse weights did not differ significantly (**Figure S5C**). Treatment with LB also significantly reduced the expression of proliferation-related proteins, including PCNA, Ki67 and C-MYC, compared with vehicle (**Figures 8C, D**). Western blotting results of PCNA protein showed a similar trend (**Figure S6**). Immunohistochemical assays of PI3K and AKT1 expression in tumor tissue showed that the percentages of cells positive for PI3K and AKT1 protein were lower in the LB than in the vehicle group (**Figures 8C, E**). Western blotting also showed that the expression of PI3K and AKT1 was lower in the LB than in the vehicle group (**Figures 8F, G**). The ratio of p-AKT1/AKT1 was significantly lower in mice administered LB than vehicle, although the p-PI3K/PI3K ratio was did not differ in the two groups (**Figure 8H**). In addition, the levels of expression of intrinsic (mitochondrial) apoptosis related molecules, such as pro-Caspase8, pro-Caspase3, pro-PARP and BCL2, were lower in tumors of mice administered LB than vehicle, whereas the levels of expression of cleaved-Caspase8, cleaved-Caspase3, cleaved-PARP and BAX were higher in tumors of mice treated with LB (**Figure 8I**). These results suggested that LB could activate intrinsic apoptosis and inhibit proliferation by downregulating the PI3K/AKT1 pathway in A549 xenograft tumors.

## Discussion

**Figure 9:**
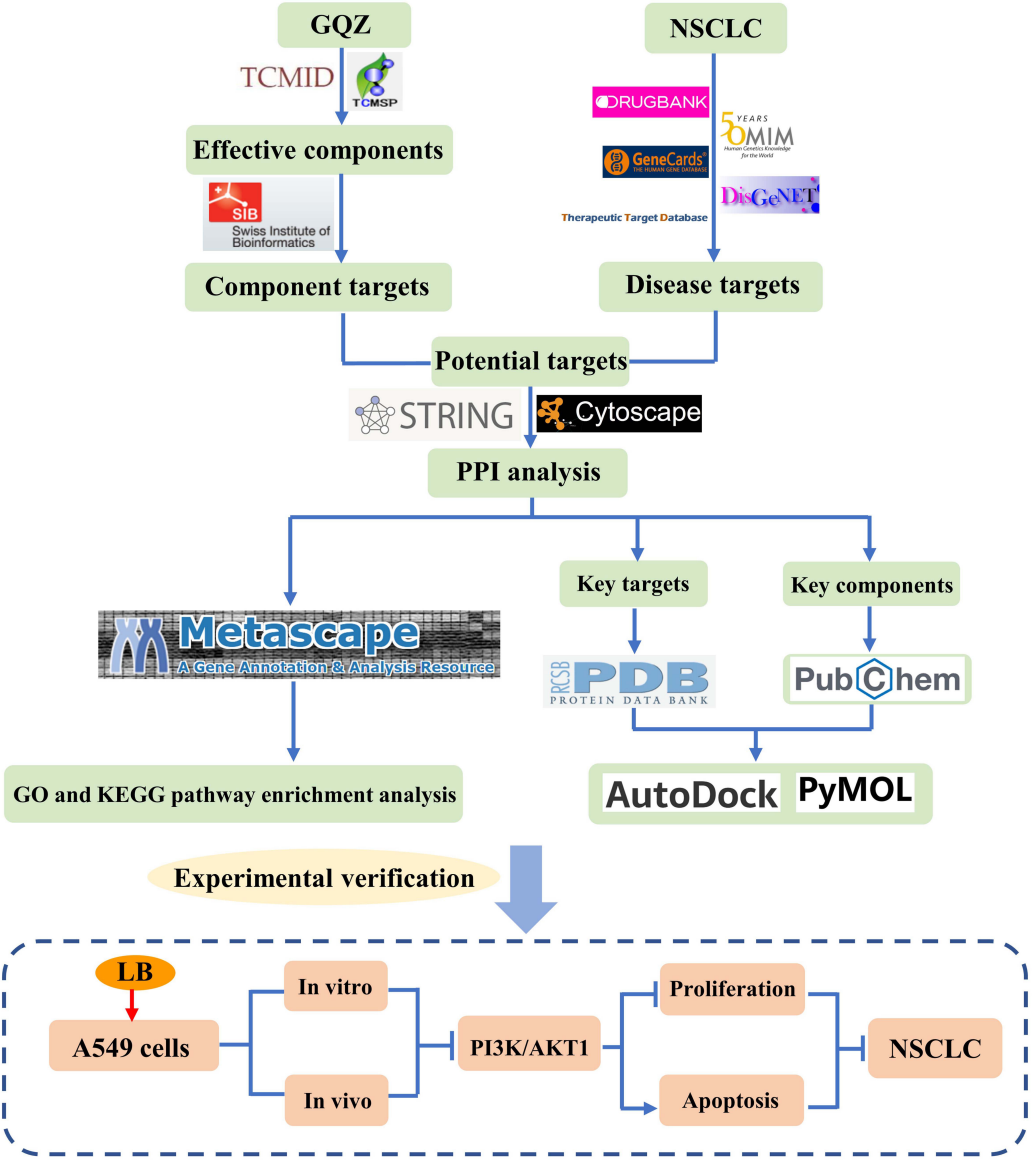
Flow chart showing the research performed in this study.

TCMs, consisting of multiple components and having multiple targets, have been used for thousands of years to prevent and treat various diseases, including diseases of the lungs (6). In recent years, natural bioactive compounds have attracted extensive attention as anticancer drugs because of their high therapeutic value and low systemic toxicity (39). For example, GQZ was reported to have therapeutic effects on kidney injury, lung injury and aging related diseases (14, 40, 41). GQZ extract enhanced the radiation of Lewis lung cancer acute hypoxic cells, although the relevant active ingredient and its mechanism of action have not yet been clearly determined (42). The present study we used comprehensive, systematic and computational pharmacological methods, as well as molecular docking technology and experimental verification to evaluate the potential molecular mechanisms of GQZ bioactive compounds in the treatment of NSCLC. The entire process of this study could be seen in **Figure 9**. LB was found to induce apoptosis and inhibit proliferation *in vitro* and *in vivo* by inhibiting the PI3K/AKT1 signaling pathway. Thus, this study provides evidence showing that LB exerts antitumor effects in A549 NSCLC cells.

Evaluation of the TCMSP, TCMID and SwissADME databases and screening by an ADME standard identified 33 active compounds in GQZ. Comparisons of common NSCLC-related targets and predicted GQZ targets identified a total of 113 predicted GQZ anti-NSCLC targets. Compound classification and GQZ compound target network analysis showed that flavonoids with good pharmacokinetic characteristics were the main active anti-NSCLC components of GQZ. These results are consistent with a previous study showing that quercetin, a flavonoid extracted from GQZ, inhibited the growth of lung tumors and induced apoptosis by upregulating pro-apoptosis and downregulating anti-apoptosis genes (43). Quercetin has been shown to have anticancer activity, inhibiting cell proliferation and metastasis and inducing apoptosis and autophagy (44–47). Quercetin was found to inhibit resistance to docetaxel in prostate cancer through the PI3K/AKT signaling pathway (48), to inhibit the AKT/CSN6/C-MYC signaling pathway and induce apoptosis of HT-29 colon cancer cells (49), and to increase the sensitivity of ovarian cancer cells to chemotherapy (50).

Construction of a PPI network allowed visualization of interactions among proteins, suggesting that AKT1, C-MYC and TP53 may play key roles in the anti-NSCLC effects of GQZ. The AKT1 and C-MYC proteins were shown to be directly related to the growth of lung cancer cells (51, 52), and TP53 was also found to affect the progression of lung cancer (53). These three target proteins were found to be highly aggregated in the cluster network, especially AKT1, which may play an important role in the process of anti-NSCLC process. GO enrichment analysis annotated the functions of GQZ protein targets, with KEGG results showing that the cancer-related signaling pathway that inhibits the enrichment of the highest gene count plays a key role in the anti NSCLC effects of GQZ. This pathway in cancer contained the PI3K/AKT1 pathway, which includes most predicted targets and focused on AKT1 (**Figure S3**). PI3K is a lipid kinase associated with the plasma membrane and consisting of three subunits, the p85 and p55 regulatory subunits and the p110 catalytic subunit. Under physiological conditions, PI3K is activated by various extracellular stimulators, such as growth factors, cytokines and hormones (54). After activation, PI3K catalyzes the phosphorylation of PtdIns (4, 5) P2 (PIP2) to produce PtdIns (3–5), P3 (PIP3), which, as a messenger, binds a subset of the downstream targets with pleckstrin homology (PH), including FYVE, Phox (PX), C1, C2 and other lipid binding domains on the cell membrane. Various signal proteins, including the kinases AKT and PDK1, can bind to the lipid products of PI3K on the cell membrane, simultaneously activate cell growth and cell survival pathways (55). Members of AKT family regulate biological functions, including cell survival, proliferation, metabolism and growth, thus affecting the progress of cancer. AKT consists of three conserved domains, an N-terminal PH domain, a central kinase catalytic domain and a C-terminal regulatory domain. The PH domain is the docking site of phosphatidylinositol 3,4,5-triphosphate (PIP3) and phosphatidylinositol 3,4-diphosphate (PIP2). AKT can be activated by various types of stimuli, including growth factors, cytokines, hormones and stress. AKT activity is strictly regulated by post-translational modifications, such as phosphorylation, ubiquitination, pantothenation, acetylation and palmitoylation (56). AKT participates in the treatment of many cancers through various signaling pathways, especially the PI3K/AKT signaling pathway. Moreover, the PI3K/AKT signaling pathway is involved in the cell cycle, as well as in cell proliferation, growth, migration, angiogenesis and apoptosis, as well as being one of the most frequently activated signal transduction pathways in cancer (57–60). Quercetin has been shown to suppress the growth of breast cancer stem cells through the PI3K/AKT signaling pathway (61). Moreover, N’-[(3Z)-1-(1-hexyl)-2-oxo-1,2-dihydro-3H-indol-3-ylidene] benzohydrazide (MDA19) was found to downregulate the expression of the main proteins involved in the EMT process, specifically PI3K/AKT/mTOR, inhibiting EMT in osteosarcoma cells (62). The PI3K/AKT pathway was also found to be activated in chronic lymphocytic leukemia (CLL) (63), high-risk myelodysplastic syndrome (MDS) and multiple myeloma (MM) (64–66). Experimental results in the present study showed that LB inhibited the levels of the expression of PI3K and AKT1 in A549 cells, both *in vitro* and *in vivo*.

C-MYC and TP53 are located downstream of AKT and are key regulators of cell growth and proliferation (67). C-MYC and TP53 are dysregulated in various cancer types (68, 69) and play critical roles in lung tumorigenesis and cancer progression (70). BCL2, Caspase8 and Caspase9 are key molecules involved in mitochondrial apoptosis (71, 72), with BCL2 protein on the mitochondrial membrane playing a regulatory role in intrinsic apoptosis. These three proteins strictly control the internal apoptosis process by inducing mitochondrial outer membrane permeability. Molecular docking experiments, performed to assess the molecular mechanism of GQZ in the treatment of NSCLC, found that quercetin bound to AKT1, C-MYC and TP53, with highest affinity binding to AKT1, indicating that AKT1 may be the key target of the anti-NSCLC effect of GQZ. In addition, BCL2, Caspase8 and Caspase9 bound well to glycine protease (GQZ12) and quercetin (GQZ33), further supporting the results of network pharmacological analysis.

Cell proliferation is one of the most important features of tumor cells. PCNA and Ki67 are two kinds of cell proliferation proteins. PCNA is mainly involved in the repair and synthesis of damaged DNA and in the regulation of the cell cycle (73). Ki67 is also involved in cell cycle regulation and acts as a marker differentiating normal and tumor cells (74). The levels of expression of both proteins are altered during the cell cycle, with large amounts of PCNA synthesized during middle and late G1 phase and high expression during S phase. Its level of expression decreased gradually during G2 phase, with the level of expression being low in M phase (75). Ki67 is almost completely unexpressed during early G1 phase, but its level gradually increases during late G1, S and G2 phases, and peaks during M phase (76). PCNA and Ki67 were reported to be significantly associated with NSCLC proliferation, which is of great significance in evaluating the occurrence, development and prognosis of NSCLC (77, 78). C-MYC is another specific marker of tumor cell proliferation. The present study found that LB could inhibit the growth of A549 subcutaneous tumors and the viability of A549 cells, indicating that LB can play an anti-tumor role in A549 cells. The levels of expression of Ki67, C-MYC and PCNA were found to be lower treated with LB than with vehicle, suggesting that LB effectively inhibited the proliferation of A549 cells *in vitro* and *in vivo*.

Apoptosis is an important manifestation of cell death, with apoptosis suppression being associated with tumor development. This signal transduction cascade was initially found during genetic screening of Caenorhabditis elegans. Mitochondrial mediated apoptosis, one of the main pathways of cell apoptosis, includes the dissipation of mitochondrial cross potential, the release of pro-apoptotic proteins into the cytoplasm, and the release of mitochondrial derived Caspase activators. Following extracellular or intracellular signal stimulation, cytochrome C is released from the permeable mitochondrial membrane into the cytoplasm. The Caspase protease front domain participates in pro-Caspase activation and downstream regulation of Caspase cascade reactions through protein-protein interactions. The core of these interactions consists of the folding of the death domain, composed of the death effector domains of pro-Caspase8 and pro-Caspase10 and the recruitment domains of pro-Caspase2 and pro-Caspase9 (79). Cytochrome C combines with apoptosis protease activating factor-1 (Apaf-1) in the cytoplasm to recruit Caspase9, form apoptotic bodies, activate Caspase9, and subsequently activate Caspase3. Activated Caspase3 can specifically lyse substrate proteins, inhibit the PARP-associated repair of DNA damage, and break DNA, ultimately leading to apoptosis (80). Proteins of the BCL2 family play important roles in regulating the mitochondria-mediated apoptotic pathway. BAX and BCL2 are the main factors controlling apoptosis (81). BAX can activate or inhibit BCL-XL and BAD, whereas BCL2 can inhibit BAX. The BAX/BCL2 activity ratio, not the level of individual proteins, is a key determinant of apoptosis indicating that this ratio is a marker of susceptibility to apoptosis (82). Flow cytometry results in the present study showed that LB promoted the apoptosis of A549 cells; the proteins related to mitochondrial apoptosis, such as pro-Caspase8, BCL2, pro-Caspase3 and pro-PARP were decreased significantly; and that the expression of BAX, cleaved-Caspase8, cleaved-Caspase3, and cleaved-PARP proteins and the BAX/BCL2 activity ratio were increased, both *in vivo* and *in vitro*. These results indicated that LB-induced cell death is under the control of mitochondrial apoptosis and the BAX/BCL2 ratio.

The present study also investigated the mechanism involved in the inhibition of A549 cells. Previous studies have demonstrated that the PI3K/AKT signaling pathway is involved in various cell biological processes, such as cell proliferation and apoptosis, especially in cancers (83). Therefore, inhibition of PI3K signaling is considered a very promising approach to the treatment of these diseases and a major target for drug development (84). LBP, one of the most important compounds of LB, was found to inhibit the proliferation and induce the apoptosis of infantile hemangioma endothelial cells by downregulating the PI3K/AKT signaling pathway (85). Although the ability of LB to affect the PI3K/AKT signaling pathway in NSCLC was unclear, the present study indicated that LB inhibited this pathway, especially downregulating the levels of AKT1 phosphorylation, consistent with the results PPI clustering analysis. Inhibition of AKT promoting apoptosis and activation of AKT attenuating apoptosis have been observed in several studies (86, 87). LB showed greater inhibition of AKT than of PI3K phosphorylation in A549 cells. In many cell lines, especially tumor cell lines, AKT function is inhibited primarily by inhibiting PI3K function, such as by treatment with specific inhibitors of PI3K, such as LY294002 and Wortmannin, as indicated by reduced p-AKT (Ser473) expression. LB markedly increased the expression of intrinsic apoptosis-related proteins and the BAX/BCL2 ratio, while reducing the expression of proliferation-related molecules, such as including BrdU, Ki67, PCNA and C-MYC. These findings indicated that LB activated apoptosis and inhibited proliferation by inhibiting the PI3K/AKT1 pathway, with the experimental results being consistent with the results of network pharmacology and molecular docking.

## Conclusion

Network pharmacology and molecular docking showed that GQZ can effectively treat NSCLC by inhibiting the PI3K/AKT1 signaling pathway, inhibiting cell proliferation through C-MYC and PCNA, and promoting apoptosis through the intrinsic pathway. Experimental validation showed that LB induced changes in relevant indicators in NSCLC were consistent with the results of network pharmacology and molecular docking. These results indicated that network pharmacology is effective in screening for drug targets in the treatment of diseases.

## Data availability statement

The datasets presented in this study can be found in online repositories. The names of the repository/repositories and accession number(s) can be found in the article/**Supplementary Material**.

## Ethics statement

The animal study was reviewed and approved by Experimental Animal Ethics Committee of Chengdu University of Traditional Chinese Medicine (ethics approval number: 2020-124).

## Author contributions

LLZ and YJG contributed to the conception and design of the review. LZ, BL, HX, WMH, ZJ, XW, XBC, and ML performed the assays. LQS and YPS assisted with data analysis. MJL and YH wrote the first draft of the manuscript. YW and LY revised the manuscript. All authors contributed to the article and approved the submitted version.

## Supplementary material

Supplementary Figure 1 Method of obtaining the *Lycium barbarum* (LB) extract and assays of its purity.

Supplementary Figure 2 Original PPI network of the NSCLC targets of GQZ obtained from the STRING database.

Supplementary Figure 3 PI3K/AKT signaling pathway adapted from the KEGG (ID: hsa04151). The targets in this pathway are shown in red.

Supplementary Figure 4 Effects of LB on expression of BCL2 and Bim mRNAs. A549 cells were treated with LB (80 μg/ml) or vehicle for 24 h, and the levels of BCL2 and Bim mRNAs were analyzed by qRT-PCR, with GAPDH mRNA used as the internal control.

Supplementary Figure 5 Effects of LB (10 mg/kg/day) on A549 xenograft tumor growth and mouse body weights. **(A)** Representative pictures of tumors *in vivo*. **(B)** Weights of xenograft tumors after 16 days of LB injection. **(C)** Body weight-time curve in mice.

Supplementary Figure 6 Effects of LB on the expression level of PCNA protein in A549 xenograft tumors, as determined by western blotting.

## Glossary

**Table:**

ABC	Avidin-Biotin-Peroxidase Complex
ADC	adenocarcinoma
AKT1	Protein kinase B
BAX	BCL2-Associated X
BCL2	B cell lymphoma/lewkmia-2
Bim	Bcl-2 interacting mediator of cell death
BrdU	5-bromo-2′-deoxyuridine
CCK8	Cell Counting Kit-8
cDNA	complementary DNA
DAB	3,3’-Diaminobenzidine
FBS	fetal bovine serum
GQZ	Gou Qi Zi
IHC	immunohistochemistry
LB	Lycium barbarum
LCC	large cell carcinoma
NSCLC	non-small cell lung cancer
OB	oral bioavailability
OMIM	Online Mendelian Inheritance in Man®
PBS	phosphate-buffered saline
PARP	poly ADP-ribose polymerase
PCNA	proliferating cell nuclear antigen
PI3K	phosphoinositide 3-kinase
PVDF	polyvinylidene fluoride
qRT-PCR	quantitative real-time polymerase chain reaction
RIPA	radio immunoprecipitation assay
SCC	squamous cell carcinoma
SDS-PAGE	sodium dodecylsulfate polyacrylamide gel electrophoresis
TCM	traditional Chinese medicine
TCMID	Traditional Chinese Medicine Information Database
TCMSP	Traditional Chinese Medicine System Pharmacology Database
TP53	Tumor protein 53
TTD	Therapeutic Target Database
WB	western blot
