# Supplementary material for: Gou Qi Zi inhibits proliferation and induces apoptosis through the PI3K/AKT1 signaling pathway in non-small cell lung cancer
Source: Front Oncol. 2022 Dec 14;12:1034750. doi: 10.3389/fonc.2022.1034750 (PMC9796997; doi:10.3389/fonc.2022.1034750)
Supplement: Supplementary file 1 [file DataSheet_1.zip › original data/Original data in Experimental verification.pdf]

Figure 7A

Sample number	OD value
Vehicle1	2.263324
Vehicle2	1.797626
Vehicle3	2.178896
Vehicle4	1.812356
Vehicle5	2.021597
Vehicle6	2.315489
LB1	0.936757
LB2	1.410657
LB3	1.002978
LB4	1.302459
LB5	1.124659
LB6	0.864916

Figure 7B

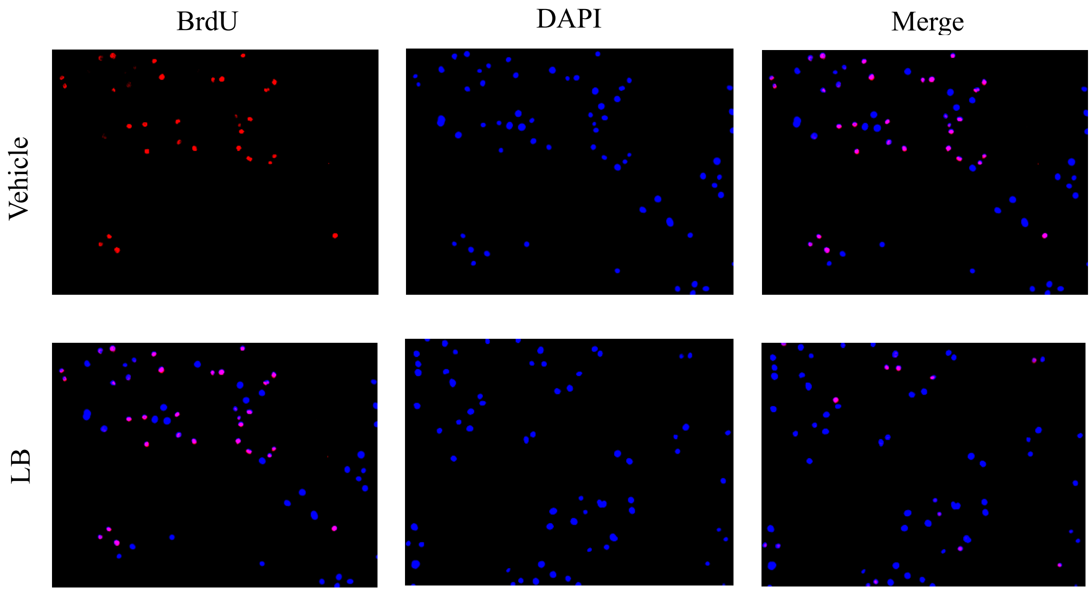

Figure 7C

Sample number	BrdU positive cells (%)
Vehicle1	53.42
Vehicle2	46.81
Vehicle3	40.71
Vehicle4	57.25
Vehicle5	43.92
Vehicle6	55.27
LB1	20.03
LB2	24.35
LB3	15.81
LB4	19.82
LB5	22.53
LB6	25.09

Figure 7D

Well	Target	Content	Sample	Cq	Cq mean	Cq Std. Dev
A01	GAPDH	Unkn-01	Vehicle1	20.85	17.59	4.617
A02	GAPDH	Unkn-01	Vehicle2	14.32	17.59	4.617
A03	GAPDH	Unkn-02	Vehicle3	15.23	14.59	0.918
A04	GAPDH	Unkn-02	Vehicle4	13.94	14.59	0.918
A05	GAPDH	Unkn-03	Vehicle5	15.69	15.37	0.455
A06	GAPDH	Unkn-03	Vehicle6	15.05	15.37	0.455
A07	GAPDH	Unkn-04	LB1	15.80	15.63	0.246
A08	GAPDH	Unkn-04	LB2	15.46	15.63	0.246
A09	GAPDH	Unkn-05	LB3	16.53	16.29	0.337
A10	GAPDH	Unkn-05	LB4	16.05	16.29	0.337
A11	GAPDH	Unkn-06	LB5	16.51	16.47	0.050
A12	GAPDH	Unkn-06	LB6	16.43	16.47	0.050
B01	PCNA	Unkn-07	Vehicle1	29.97	28.38	0.158
B02	PCNA	Unkn-07	Vehicle2	29.99	28.38	0.158
B03	PCNA	Unkn-08	Vehicle3	27.50	29.84	0.234
B04	PCNA	Unkn-08	Vehicle4	27.57	29.84	0.234
B05	PCNA	Unkn-09	Vehicle5	27.99	30.19	0.139
B06	PCNA	Unkn-09	Vehicle6	28.09	30.19	0.139
B07	PCNA	Unkn-10	LB1	30.44	36.43	1.402
B08	PCNA	Unkn-10	LB2	30.42	36.43	1.402
B09	PCNA	Unkn-11	LB3	30.97	30.95	0.033
B10	PCNA	Unkn-11	LB4	30.92	30.95	0.033
B11	PCNA	Unkn-12	LB5	30.83	29.36	0.098
B12	PCNA	Unkn-12	LB6	30.89	29.36	0.098

Figure 7E

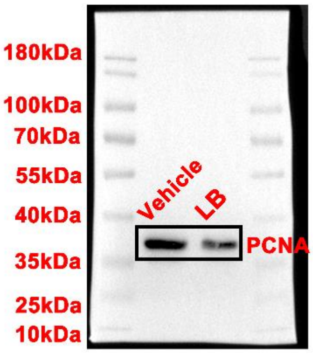
180kDa
100kDa
70kDa
55kDa
40kDa
35kDa
25kDa
10kDa
Vehicle
LB
PCNA

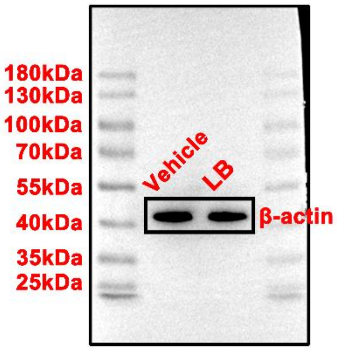
180kDa
130kDa
100kDa
70kDa
55kDa
40kDa
35kDa
25kDa
Vehicle
LB
β-actin

Figure 7F

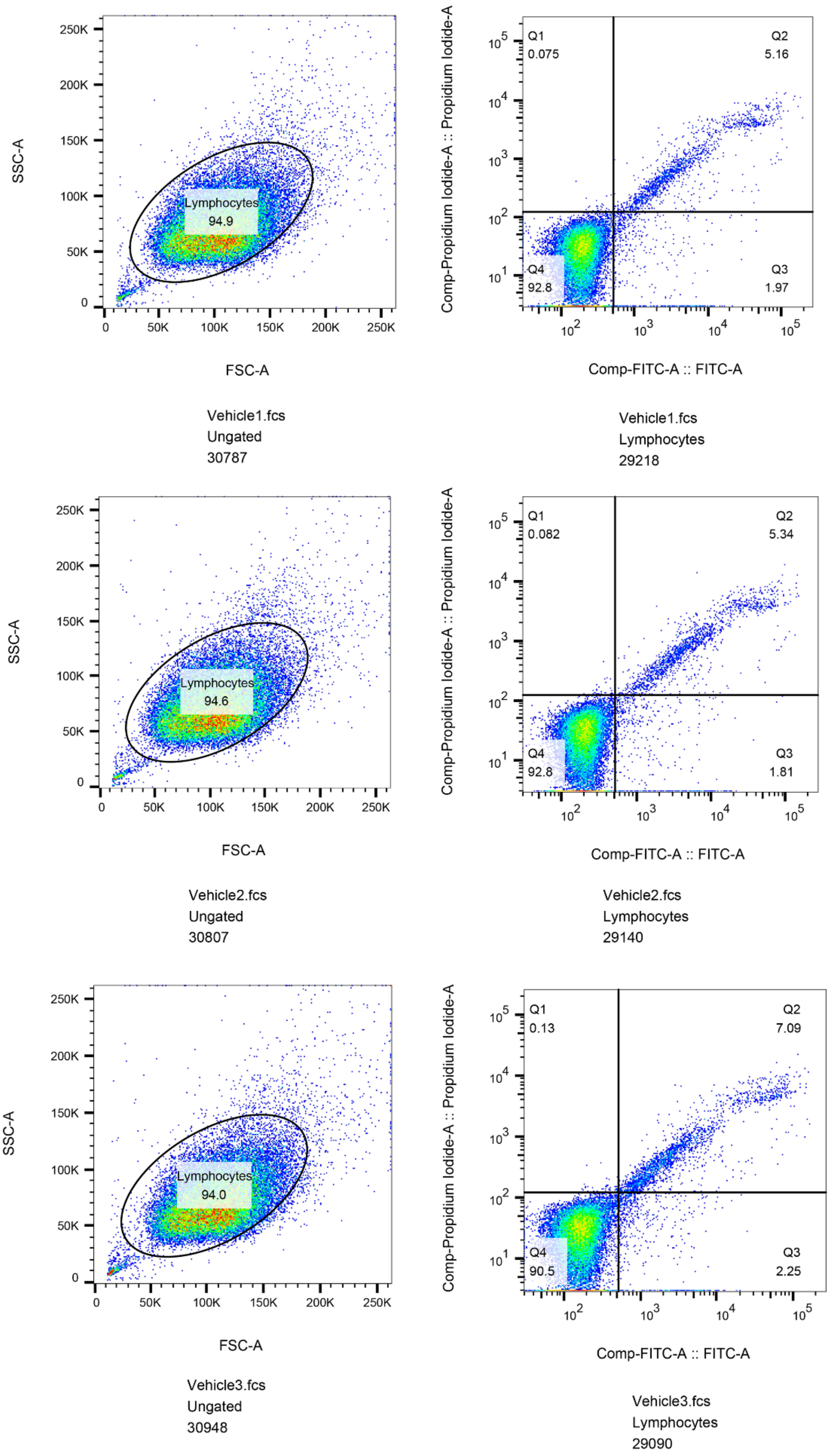

SSC-A
FSC-A
Lymphocytes
94.9
Vehicle1.fcs
Ungated
30787
Comp-Propidium Iodide-A :: Propidium Iodide-A
Comp-FITC-A :: FITC-A
Q1
0.075
Q2
5.16
Q3
1.97
Q4
92.8
Vehicle1.fcs
Lymphocytes
29218
Lymphocytes
94.6
Vehicle2.fcs
Ungated
30807
Q1
0.082
Q2
5.34
Q3
1.81
Q4
92.8
Vehicle2.fcs
Lymphocytes
29140
Lymphocytes
94.0
Vehicle3.fcs
Ungated
30948
Q1
0.13
Q2
7.09
Q3
2.25
Q4
90.5
Vehicle3.fcs
Lymphocytes
29090

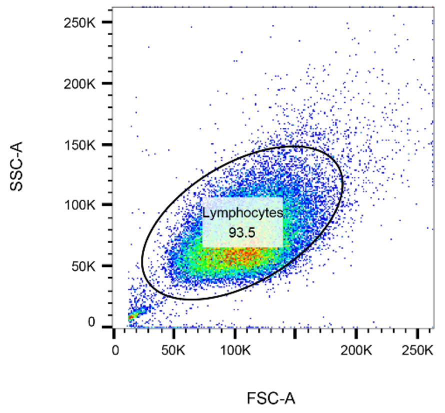

Vehicle4.fcs
Ungated
31112

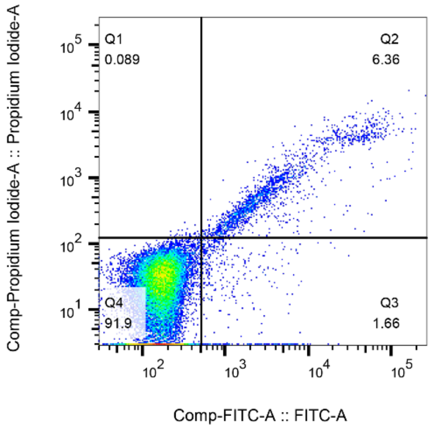

Vehicle4.fcs
Lymphocytes
29091

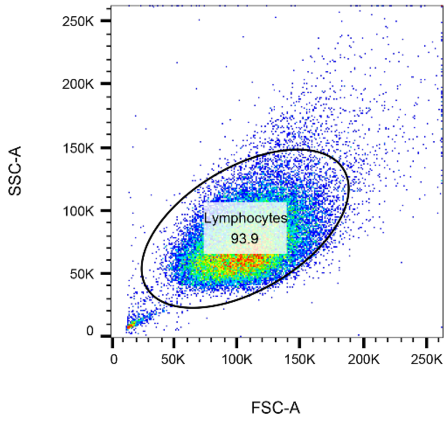

Vehicle5.fcs
Ungated
30952

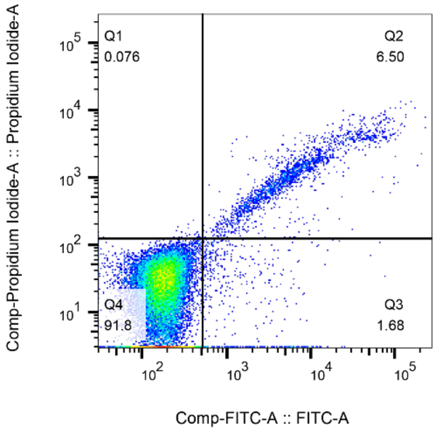

Vehicle5.fcs
Lymphocytes
29062

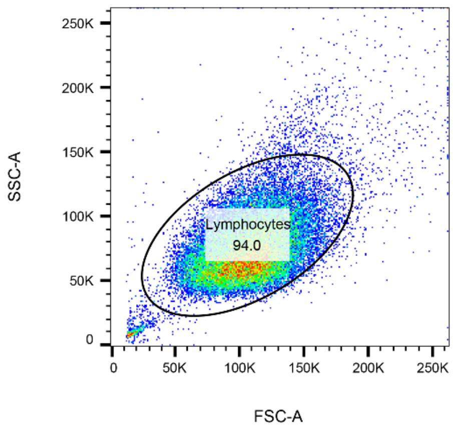

Vehicle6.fcs
Ungated
30978

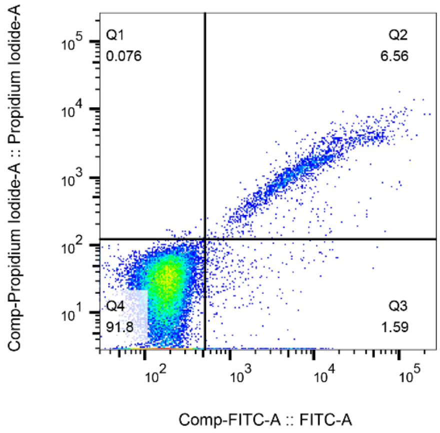

Vehicle6.fcs
Lymphocytes
29109

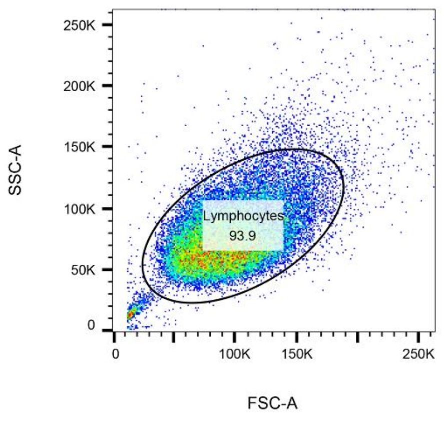

LB1.fcs
Ungated
30985

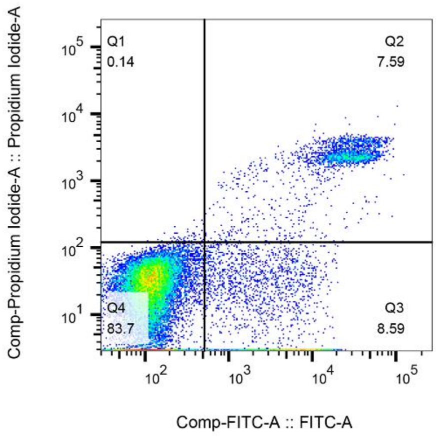

LB1.fcs
Lymphocytes
29103

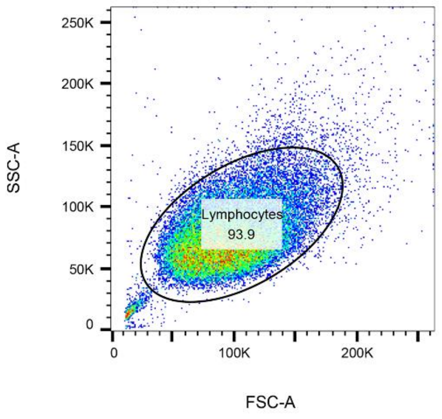

LB2.fcs
Ungated
30995

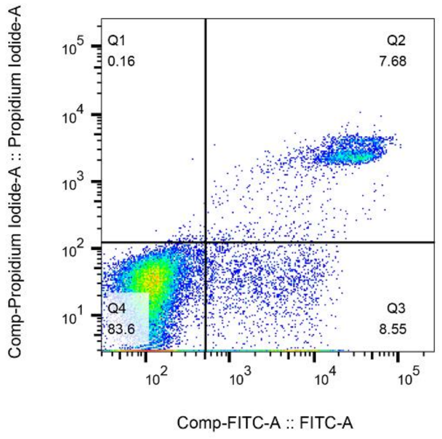

LB2.fcs
Lymphocytes
29102

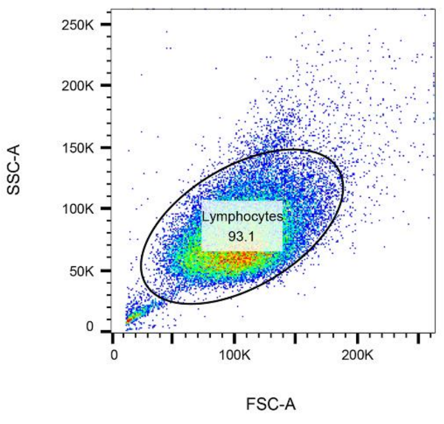

LB3.fcs
Ungated
31194

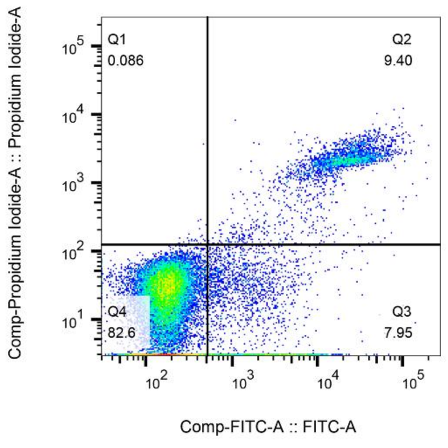

LB3.fcs
Lymphocytes
29054

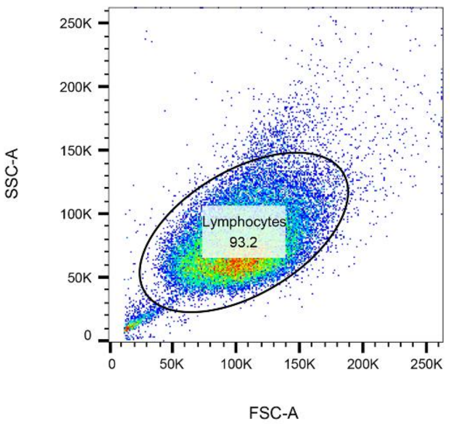

LB4.fcs
Ungated
31184

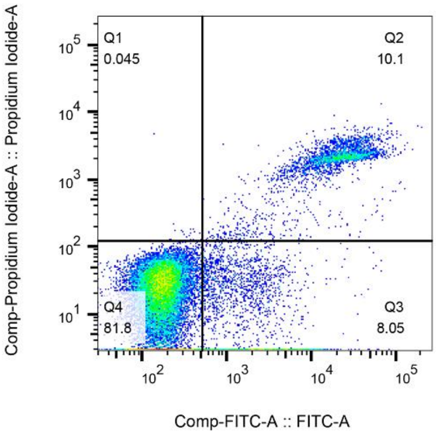

LB4.fcs
Lymphocytes
29059

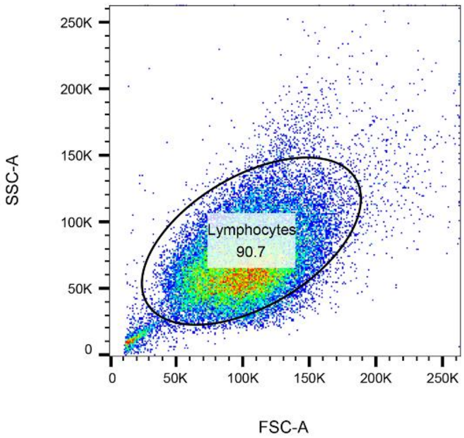

LB5.fcs
Ungated
31886

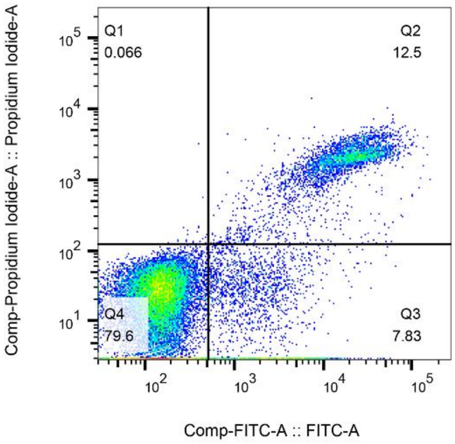

LB5.fcs
Lymphocytes
28935

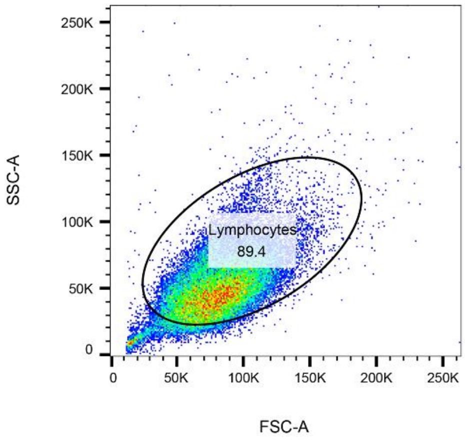

LB6.fcs
Ungated
34624

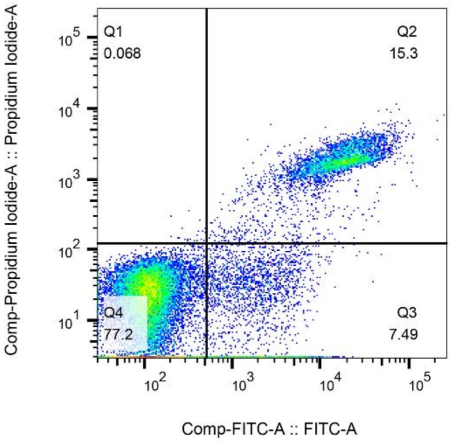

LB6.fcs
Lymphocytes
30949

Figure 7G

Sample number	Rate of cell apoptosis (%)
Vehicle1	7.13
Vehicle2	7.15
Vehicle3	9.34
Vehicle4	8.02
Vehicle5	8.18
Vehicle6	8.15
LB1	16.18
LB2	16.23
LB3	17.35
LB4	18.15
LB5	20.33
LB6	22.79

Figure 7H

Well	Target	Content	Sample	Cq	Cq mean	Cq Std. Dev
A01	GAPDH	Unkn-01	Vehicle1	17.14	16.94	0.283
A02	GAPDH	Unkn-01	Vehicle2	16.74	16.94	0.283
A03	GAPDH	Unkn-02	Vehicle3	17.68	17.66	0.033
A04	GAPDH	Unkn-02	Vehicle4	17.64	17.66	0.033
A05	GAPDH	Unkn-03	Vehicle5	18.19	18.28	0.132
A06	GAPDH	Unkn-03	Vehicle6	18.37	18.28	0.132
A07	GAPDH	Unkn-04	LB1	20.26	20.38	0.165
A08	GAPDH	Unkn-04	LB2	20.50	20.38	0.165

A09	GAPDH	Unkn-05	LB3	18.60	18.95	0.497
A10	GAPDH	Unkn-05	LB4	19.30	18.95	0.497
A11	GAPDH	Unkn-06	LB5	17.04	16.91	0.196
A12	GAPDH	Unkn-06	LB6	16.77	16.91	0.196
B01	PI3K	Unkn-07	Vehicle1	25.23	25.13	0.141
B02	PI3K	Unkn-07	Vehicle2	25.03	25.13	0.141
B03	PI3K	Unkn-08	Vehicle3	26.42	26.37	0.066
B04	PI3K	Unkn-08	Vehicle4	26.33	26.37	0.066
B05	PI3K	Unkn-09	Vehicle5	27.20	27.01	0.266
B06	PI3K	Unkn-09	Vehicle6	26.83	27.01	0.266
B07	PI3K	Unkn-10	LB1	29.26	22.34	0.113
B08	PI3K	Unkn-10	LB2	29.42	22.34	0.113
B09	PI3K	Unkn-11	LB3	29.06	23.11	0.074
B10	PI3K	Unkn-11	LB4	29.16	23.11	0.074
B11	PI3K	Unkn-12	LB5	26.74	24.67	0.107
B12	PI3K	Unkn-12	LB6	26.59	24.67	0.107
C01	AKT1	Unkn-13	Vehicle1	23.10	23.11	0.023
C 02	AKT1	Unkn-13	Vehicle2	23.13	23.11	0.023
C 03	AKT1	Unkn-14	Vehicle3	24.18	24.18	0.006
C 04	AKT1	Unkn-14	Vehicle4	24.19	24.18	0.006
C 05	AKT1	Unkn-15	Vehicle5	24.65	24.71	0.093
C 06	AKT1	Unkn-15	Vehicle6	24.78	24.71	0.093
C 07	AKT1	Unkn-16	LB1	27.91	26.92	0.007
C 08	AKT1	Unkn-40	LB2	27.92	26.92	0.007
G09	AKT1	Unkn-41	LB3	26.16	25.11	0.071
C 10	AKT1	Unkn-41	LB4	26.05	25.11	0.071
C 11	AKT1	Unkn-42	LB5	24.76	22.95	0.272
C 12	AKT1	Unkn-42	LB6	24.14	22.95	0.272

Figure 7I

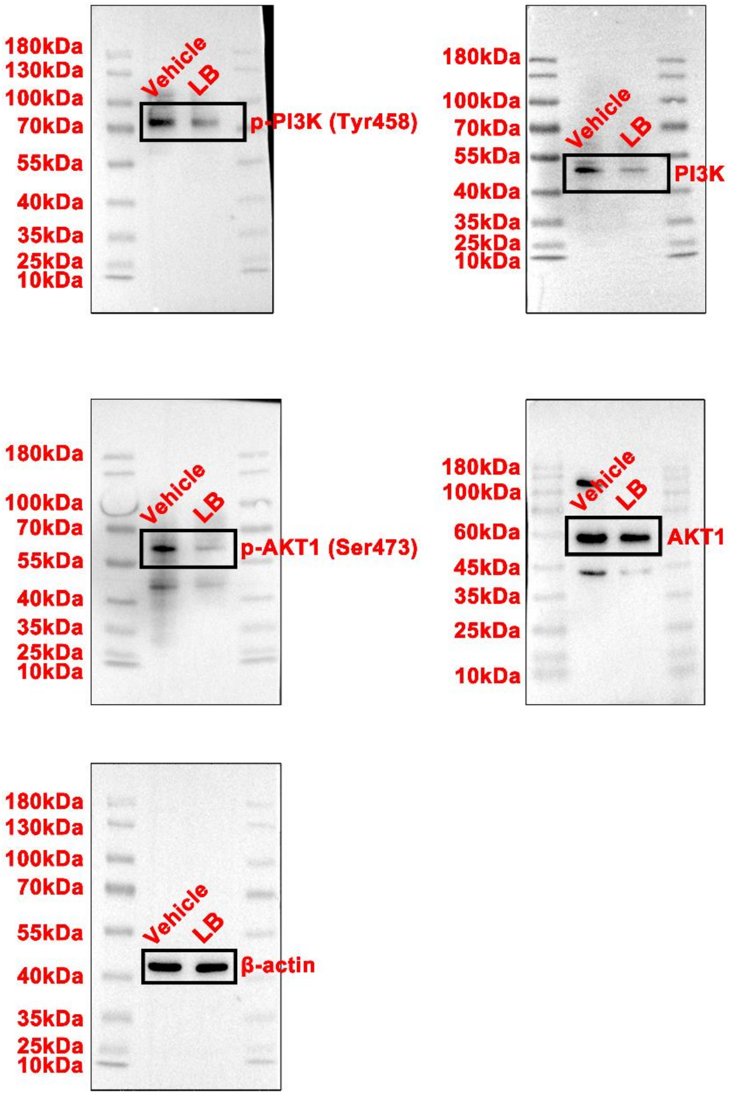

180kDa
130kDa
100kDa
70kDa
55kDa
40kDa
35kDa
25kDa
10kDa
Vehicle
LB
p-PI3K (Tyr458)
PI3K
p-AKT1 (Ser473)
AKT1
60kDa
45kDa
β-actin

Figure 7J

Sample number	Gray value	
	PI3K	AKT1
Vehicle1	1.016287	1.120773
Vehicle2	1.074919	1.198068
Vehicle3	0.840391	0.917874
Vehicle4	0.762215	0.850242
Vehicle5	1.18241	1.091787
Vehicle6	1.123779	0.821256
LB1	0.19544	0.724638
LB2	0.29316	0.772947
LB3	0.400651	0.801932
LB4	0.2443	0.695652
LB5	0.14658	0.830918
LB6	0.34202	0.753623

Figure 8K

Sample number	Gray value	
	p-PI3K (Tyr458)/PI3K	p-AKT1 (Ser473)/AKT1
Vehicle1	1.0752	0.96359743
Vehicle2	0.912	1.130620985
Vehicle3	0.864	0.822269807
Vehicle4	1.152	0.899357602
Vehicle5	0.816	1.053533191
Vehicle6	1.1808	1.130620985
LB1	1.0176	0.192719486
LB2	1.1328	0.128479657
LB3	0.8928	0.256959315
LB4	0.816	0.244111349
LB5	1.1712	0.282655246
LB6	0.912	0.334047109

Figure 8L

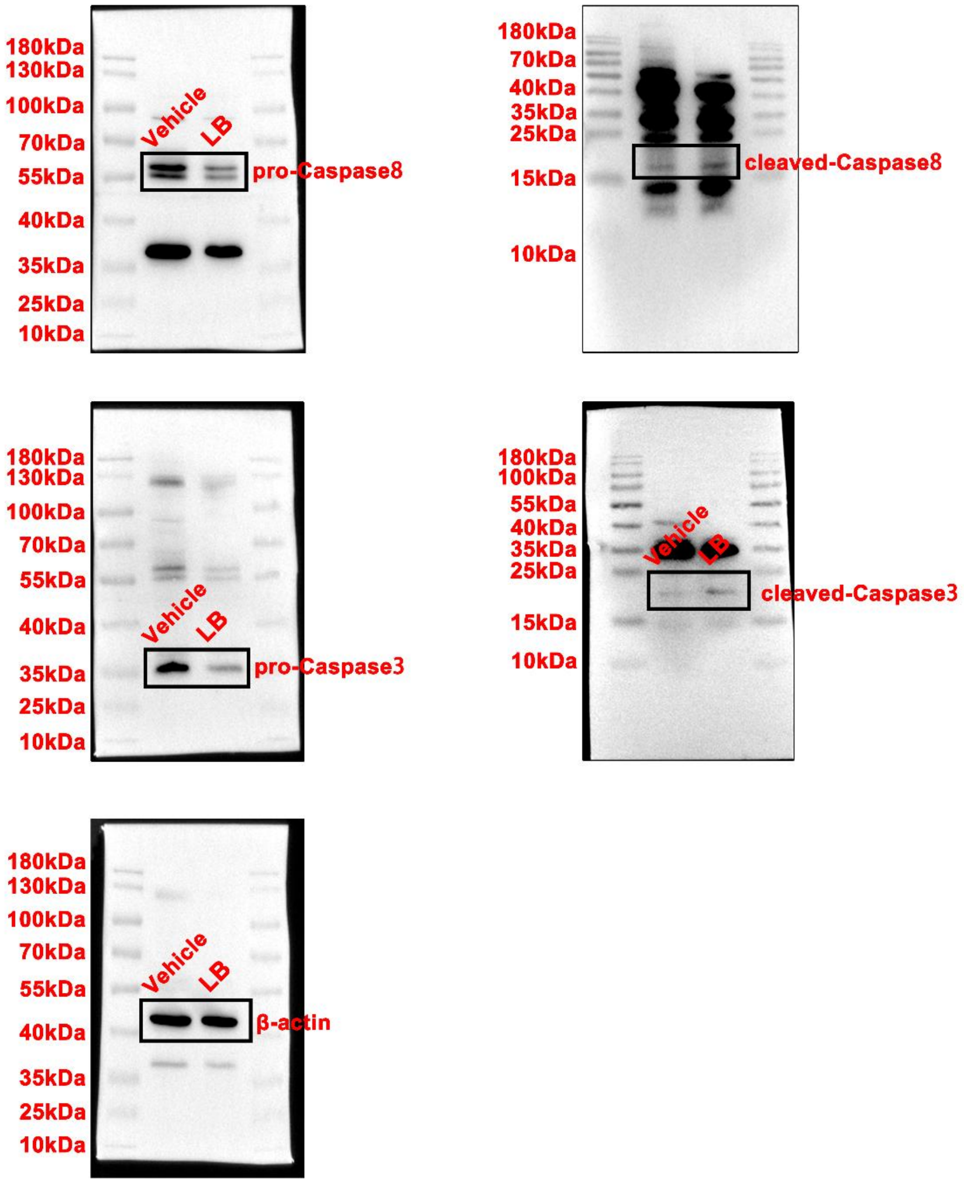
180kDa
130kDa
100kDa
70kDa
55kDa
40kDa
35kDa
25kDa
10kDa
Vehicle
LB
pro-Caspase8
180kDa
70kDa
40kDa
35kDa
25kDa
15kDa
10kDa
cleaved-Caspase8
180kDa
130kDa
100kDa
70kDa
55kDa
40kDa
35kDa
25kDa
10kDa
Vehicle
LB
pro-Caspase3
180kDa
100kDa
55kDa
40kDa
35kDa
25kDa
15kDa
10kDa
Vehicle
LB
cleaved-Caspase3
180kDa
130kDa
100kDa
70kDa
55kDa
40kDa
35kDa
25kDa
10kDa
Vehicle
LB
β-actin

Figure 8L

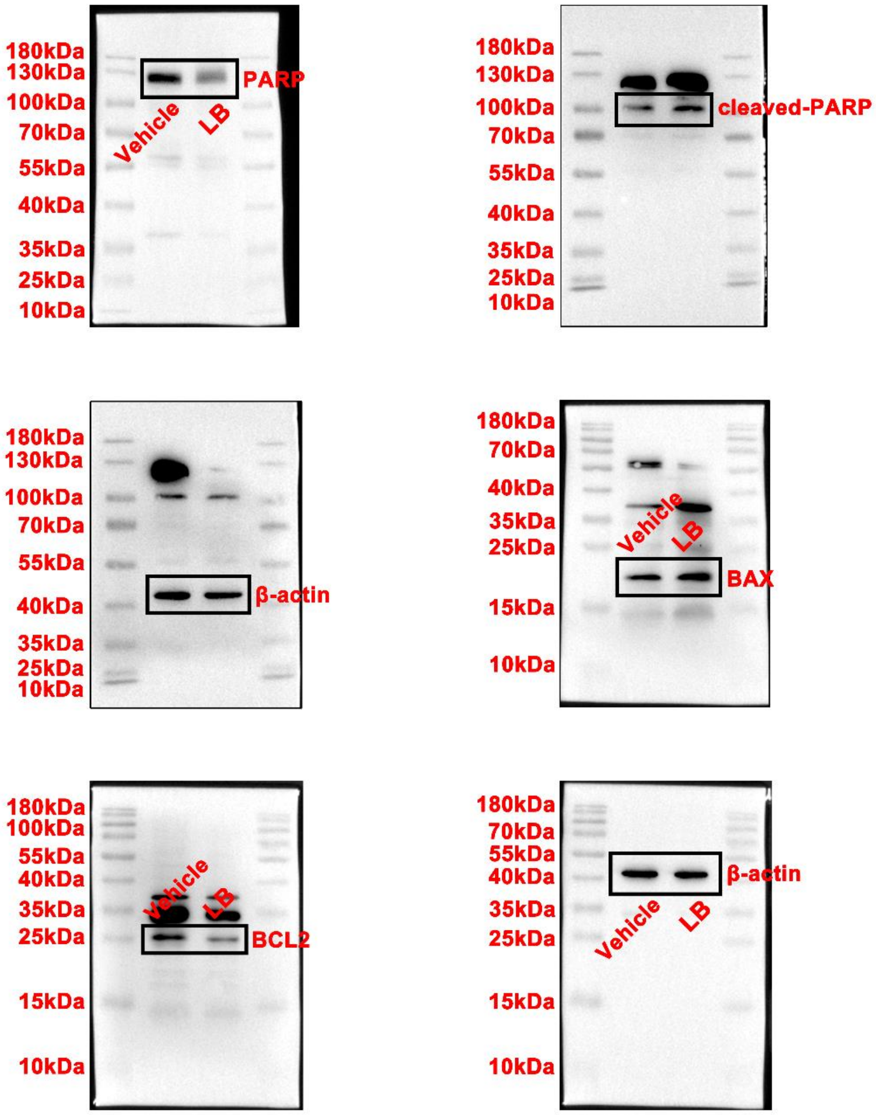
180kDa
130kDa
100kDa
70kDa
55kDa
40kDa
35kDa
25kDa
10kDa
Vehicle
LB
PARP
cleaved-PARP
β-actin
BAX
15kDa
BCL2

Figure 8A

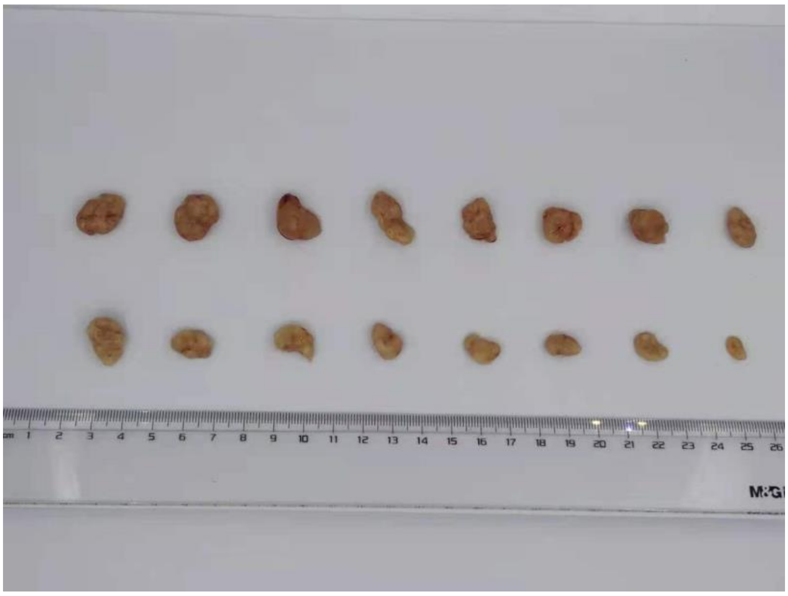

Figure 8B

Mouse number	Volume (mm^3)				
	0	4	8	12	16 (days)
Vehicle1	43.45	200.62	375.86	980.54	1463.5
Vehicle2	35.58	124.25	363.23	937.63	1416.49
Vehicle3	27.72	119.06	256.5	853.84	1379.6
Vehicle4	26.14	102.73	247.29	806.12	1108.6
Vehicle5	24.68	91.78	215.73	716.5	1106.5
Vehicle6	22.05	84.36	199.26	681.09	1071.6
Vehicle7	18	83.45	193.5	681.09	1069
Vehicle8	18	79.69	188.98	670.94	1043.1
LB1	40.56	92.27	204.19	380.54	425
LB2	40.34	46.1	67.23	307.21	409.2
LB3	39.75	45.15	53.23	267.84	319.4
LB4	31.046	39.95	51.12	202.34	300.2
LB5	30.0125	39.95	45.68	202.34	294.4
LB6	26.011	34.54	44.3	210.11	230.5
LB7	22.6	31.48	41.91	158.19	160
LB8	20.33	29.7	35.52	116.14	120.016

Figure 8C

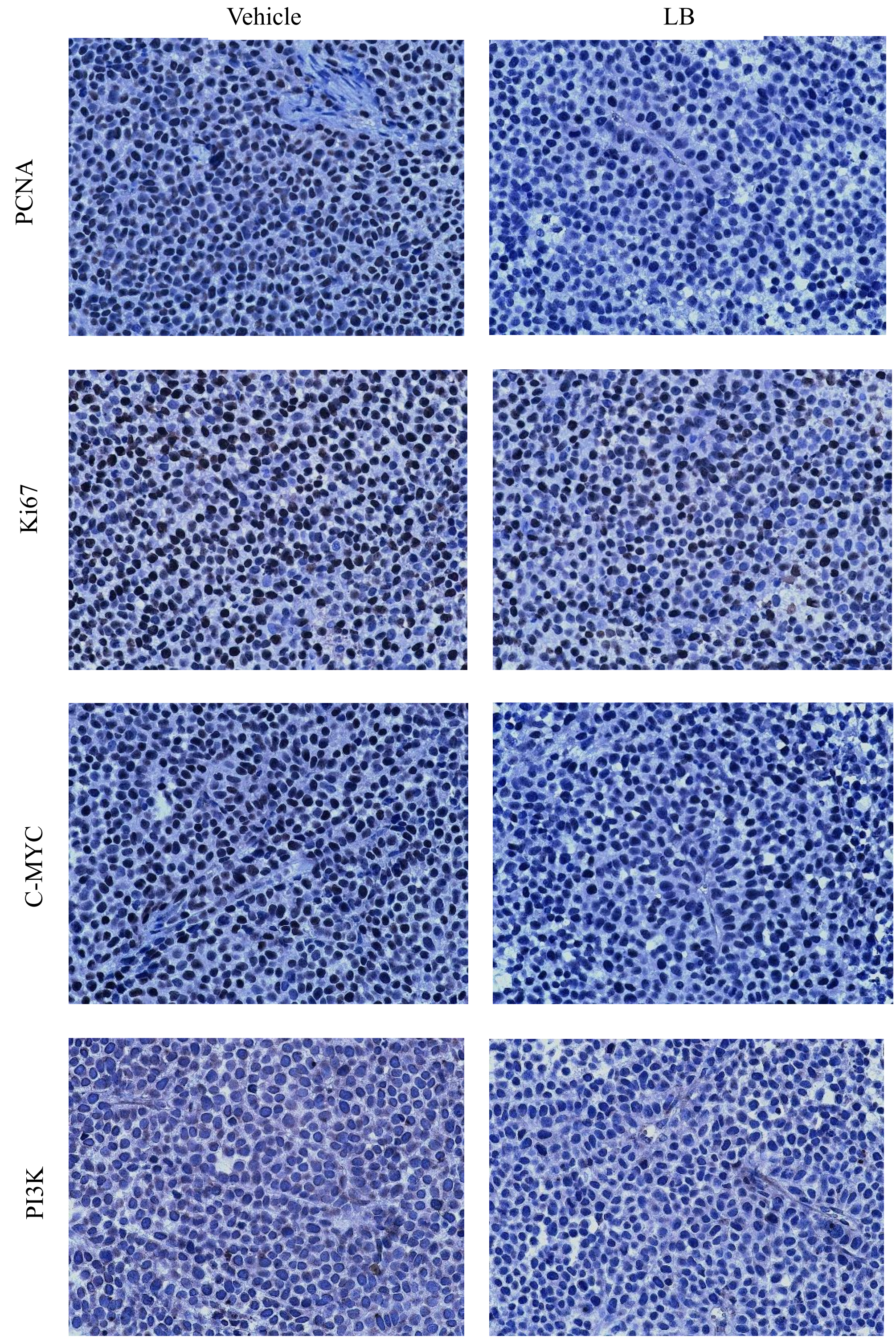

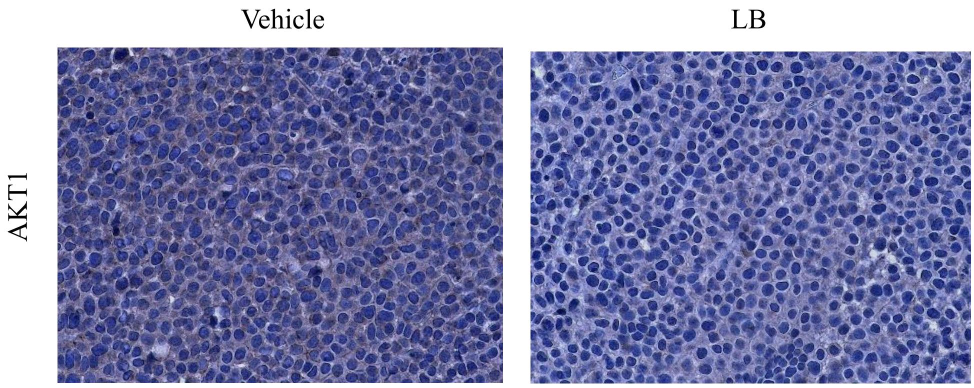

Figure 8D

Sample number	Positive staining level (%)		
	PCNA	Ki67	C-MYC
Vehicle1	48.42	65.03	78.04
Vehicle2	55.72	69.56	69.73
Vehicle3	52.34	75.35	72.25
Vehicle4	40.17	58.25	80.41
Vehicle5	58.24	55.15	75.73
Vehicle6	42.46	50.34	71.85
Vehicle7	60.56	68.73	73.35
Vehicle8	48.51	63.53	80.15
LB1	23.05	33.36	30.24
LB2	15.12	28.24	27.14
LB3	13.08	38.25	36.32
LB4	16.15	24.61	20.13
LB5	10.98	39.25	26.15
LB6	9.34	20.15	39.25
LB7	15.05	40.73	35.25
LB8	11.35	36.82	33.92

Figure 8E

Sample number	Positive staining level (%)	
	PI3K	AKT1
Vehicle1	40.53	73.63
Vehicle2	50.69	53.65
Vehicle3	44.83	63.02
Vehicle4	39.26	50.67
Vehicle5	56.35	70.1
Vehicle6	49.62	67.33
Vehicle7	42.16	55.93
Vehicle8	53.86	63.67
LB1	20.06	30.02
LB2	15.87	42.36
LB3	22.33	28.78
LB4	19.86	38.43
LB5	26.56	22.08
LB6	23.02	35.34
LB7	18.56	28.43
LB8	27.93	35.23

Figure 8F

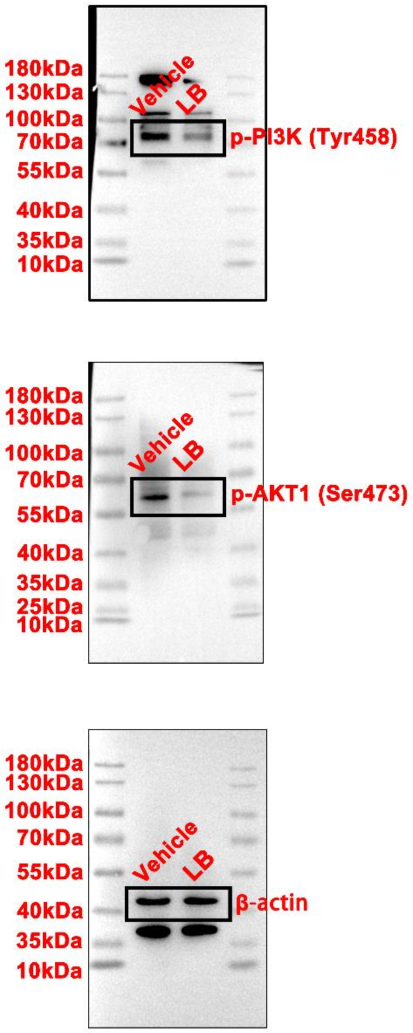
180kDa
130kDa
100kDa
70kDa
55kDa
40kDa
35kDa
10kDa
Vehicle
LB
p-PI3K (Tyr458)
180kDa
100kDa
70kDa
55kDa
40kDa
35kDa
25kDa
10kDa
Vehicle
LB
PI3K
180kDa
130kDa
100kDa
70kDa
55kDa
40kDa
35kDa
25kDa
10kDa
Vehicle
LB
p-AKT1 (Ser473)
180kDa
130kDa
100kDa
70kDa
55kDa
40kDa
35kDa
25kDa
10kDa
Vehicle
LB
AKT1
180kDa
130kDa
100kDa
70kDa
55kDa
40kDa
35kDa
10kDa
Vehicle
LB
β-actin

Figure 8G

Sample number	Gray value	
	PI3K	AKT1
Vehicle1	1.030599026	1.058528831
Vehicle2	1.084282094	0.927983043
Vehicle3	0.855935954	1.098607833
Vehicle4	1.221772539	0.871236572
Vehicle5	0.876308485	1.189652681
Vehicle6	0.931101903	0.85399104
LB1	0.312829384	0.75587456
LB2	0.3876574	0.668914688
LB3	0.310705234	0.582687027
LB4	0.277684355	0.698068308
LB5	0.374236634	0.613420685
LB6	0.287339582	0.64897153

Figure 8H

Sample number	Gray value	
	p-PI3K (Tyr458)/PI3K	p-AKT1 (Ser473)/AKT1
Vehicle1	1.004926108	1.033846154
Vehicle2	0.906403941	0.913846154
Vehicle3	1.093596059	1.006153846
Vehicle4	0.837438424	0.867692308
Vehicle5	1.044334975	1.116923077
Vehicle6	1.113300493	1.061538462
LB1	0.926108374	0.203076923
LB2	1.004926108	0.350769231
LB3	1.044334975	0.286153846
LB4	0.945812808	0.258461538
LB5	0.926108374	0.36
LB6	1.064039409	0.230769231

Figure 8I

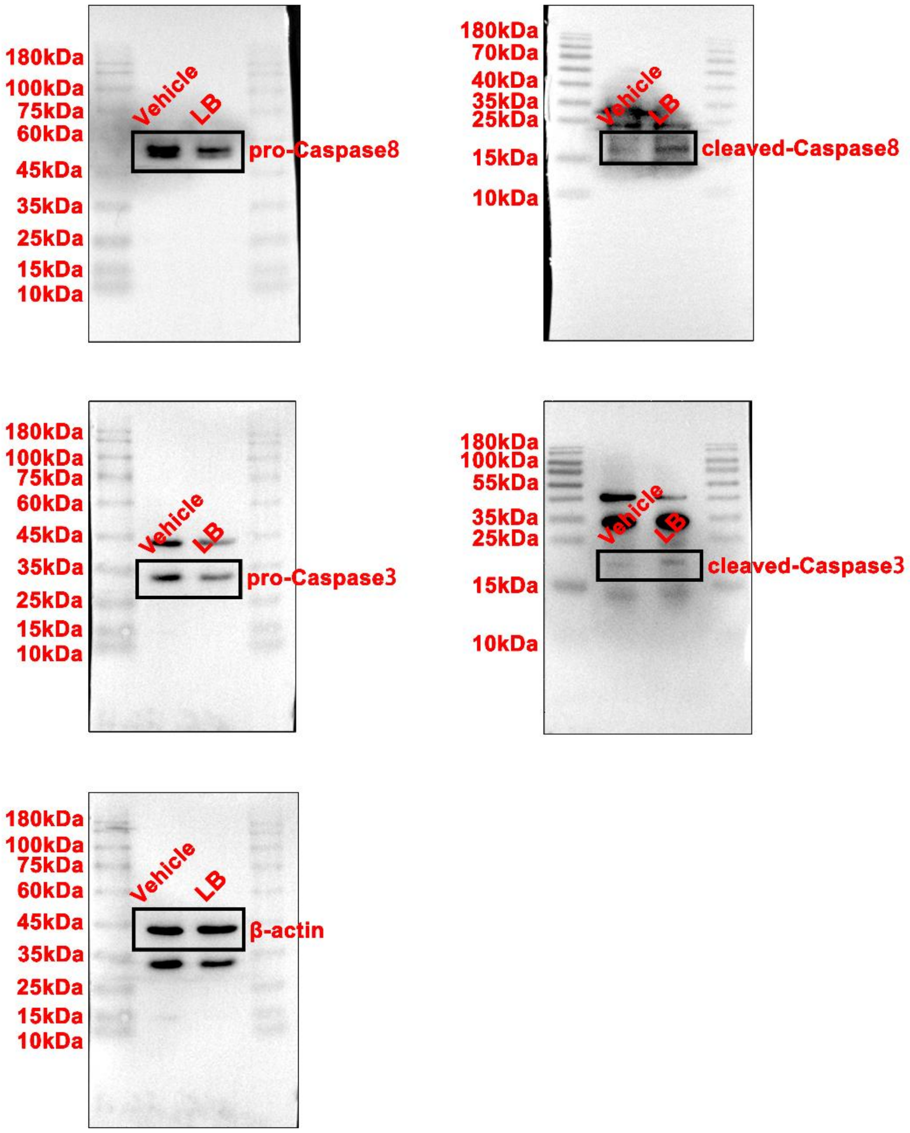
180kDa
100kDa
75kDa
60kDa
45kDa
35kDa
25kDa
15kDa
10kDa
Vehicle
LB
pro-Caspase8
180kDa
70kDa
40kDa
35kDa
25kDa
15kDa
10kDa
Vehicle
LB
cleaved-Caspase8
180kDa
100kDa
75kDa
60kDa
45kDa
35kDa
25kDa
15kDa
10kDa
Vehicle
LB
pro-Caspase3
180kDa
100kDa
55kDa
35kDa
25kDa
15kDa
10kDa
Vehicle
LB
cleaved-Caspase3
180kDa
100kDa
75kDa
60kDa
45kDa
35kDa
25kDa
15kDa
10kDa
Vehicle
LB
β-actin

Figure 8I

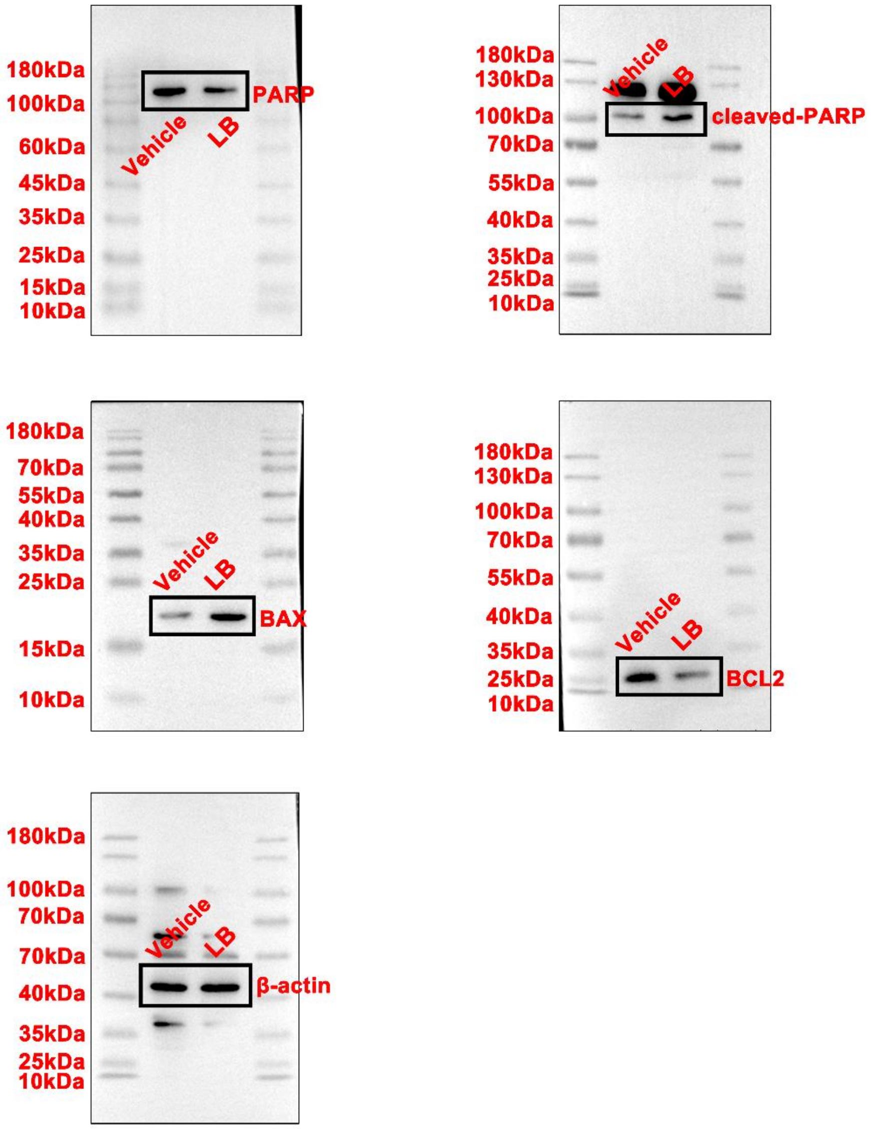
180kDa
100kDa
60kDa
45kDa
35kDa
25kDa
15kDa
10kDa
Vehicle
LB
PARP
180kDa
130kDa
100kDa
70kDa
55kDa
40kDa
35kDa
25kDa
10kDa
Vehicle
LB
cleaved-PARP
180kDa
70kDa
55kDa
40kDa
35kDa
25kDa
15kDa
10kDa
Vehicle
LB
BAX
180kDa
130kDa
100kDa
70kDa
55kDa
40kDa
35kDa
25kDa
10kDa
Vehicle
LB
BCL2
180kDa
100kDa
70kDa
70kDa
40kDa
35kDa
25kDa
10kDa
Vehicle
LB
β-actin

Figure S4

Well	Target	Content	Sample	Cq	Cq mean	Cq Std. Dev
A01	GAPDH	Unkn-01	Vehicle1	20.85	17.59	4.617
A02	GAPDH	Unkn-01	Vehicle2	14.32	17.59	4.617
A03	GAPDH	Unkn-02	Vehicle3	15.23	14.59	0.918
A04	GAPDH	Unkn-02	Vehicle4	13.94	14.59	0.918
A05	GAPDH	Unkn-03	Vehicle5	15.69	15.37	0.455
A06	GAPDH	Unkn-03	Vehicle6	15.05	15.37	0.455
A07	GAPDH	Unkn-04	LB1	15.80	15.63	0.246
A08	GAPDH	Unkn-04	LB2	15.46	15.63	0.246
A09	GAPDH	Unkn-05	LB3	16.53	16.29	0.337
A10	GAPDH	Unkn-05	LB4	16.05	16.29	0.337
A11	GAPDH	Unkn-06	LB5	16.51	16.47	0.050
A12	GAPDH	Unkn-06	LB6	16.43	16.47	0.050
C01	BCL2	Unkn-13	Vehicle1	29.97	28.38	0.158
C02	BCL2	Unkn-13	Vehicle2	29.99	28.38	0.158
C03	BCL2	Unkn-14	Vehicle3	27.50	29.84	0.234
C04	BCL2	Unkn-14	Vehicle4	27.57	29.84	0.234
C05	BCL2	Unkn-15	Vehicle5	27.99	30.19	0.139
C06	BCL2	Unkn-15	Vehicle6	28.09	30.19	0.139
C07	BCL2	Unkn-16	LB1	30.44	36.43	1.402
C08	BCL2	Unkn-16	LB2	30.42	36.43	1.402
C09	BCL2	Unkn-17	LB3	30.97	30.95	0.033
C10	BCL2	Unkn-17	LB4	30.92	30.95	0.033
C11	BCL2	Unkn-18	LB5	30.83	29.36	0.098
C12	BCL2	Unkn-18	LB6	30.89	29.36	0.098
D01	Bim	Unkn-19	Vehicle1	30.77	32.80	0.810
D02	Bim	Unkn-19	Vehicle2	30.52	32.80	0.810
D03	Bim	Unkn-20	Vehicle3	27.20	28.71	2.107
D04	Bim	Unkn-20	Vehicle4	27.22	28.71	2.107

D05	Bim	Unkn-21	Vehicle5	28.72	28.32	0.569
D06	Bim	Unkn-21	Vehicle6	27.92	28.32	0.569
D07	Bim	Unkn-22	LB1	26.38	27.21	0.088
D08	Bim	Unkn-22	LB2	26.35	27.21	0.088
D09	Bim	Unkn-23	LB3	27.19	28.02	0.044
D10	Bim	Unkn-23	LB4	27.15	28.02	0.044
D11	Bim	Unkn-24	LB5	27.17	27.71	0.086
D12	Bim	Unkn-24	LB6	27.25	27.71	0.086

Figure S5A

Vehicle LB

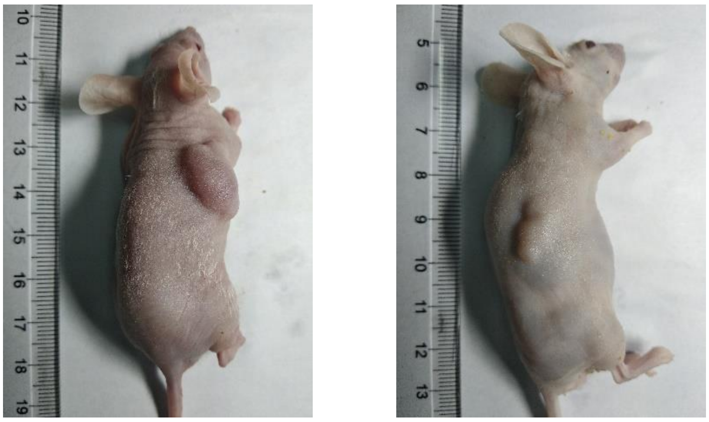

Figure S5B

Mouse number	Tumor weight (mg)
Vehicle1	1080
Vehicle2	990
Vehicle3	800
Vehicle4	800
Vehicle5	750
Vehicle6	690
Vehicle7	640
Vehicle8	580
LB1	440
LB2	430
LB3	420
LB4	330
LB5	310
LB6	200
LB7	190
LB8	100

Figure S5B

Mouse number	Body weight (g)				
	0	4	8	12	16 (days)
Vehicle1	22.5	24	24	24.6	24.7
Vehicle2	22.8	23.8	24.28	24.5	24.67
Vehicle3	22.6	23.3	23.6	23.9	24.05
Vehicle4	23.8	22	23.7	24.7	25.3
Vehicle5	22.3	23	23	23.9	24.2
Vehicle6	22	23.5	23.72	23.8	24.7
Vehicle7	22.3	23.3	23.3	24.6	24.6
Vehicle8	22.5	23	23.18	23.6	24.35
LB1	22.3	23.8	23.7	24.33	24.6
LB2	21.6	23.5	23.7	23.7	24
LB3	22.2	22.6	22.93	23.3	23.6
LB4	22.2	23.5	23.57	24	24.2
LB5	22.5	23	24.3	24.6	25.3
LB6	23	23	23.65	23.74	24.1
LB7	22.8	22.5	23.47	24.5	24.5
LB8	22.3	22.8	22.88	24	24.9

Figure S6

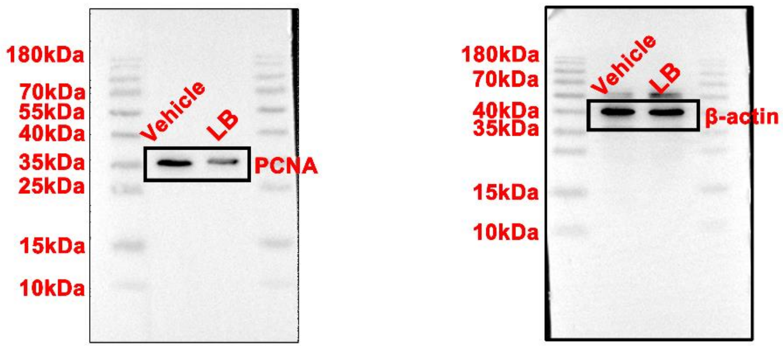
180kDa
70kDa
55kDa
40kDa
35kDa
25kDa
15kDa
10kDa
Vehicle
LB
PCNA
180kDa
70kDa
40kDa
35kDa
15kDa
10kDa
Vehicle
LB
β-actin
