# Supplementary material for: Gou Qi Zi inhibits proliferation and induces apoptosis through the PI3K/AKT1 signaling pathway in non-small cell lung cancer
Source: Front Oncol. 2022 Dec 14;12:1034750. doi: 10.3389/fonc.2022.1034750 (PMC9796997; doi:10.3389/fonc.2022.1034750)
Supplement: Supplementary Figure 1 — Method of obtaining the Lycium barbarum (LB) extract and assays of its purity. [file DataSheet_2.pdf]

1. Supplementary Tables

Table S1. List of primer sequences used for qRT-PCR in the study

Gene Name	Species	Sequence
GAPDH	Human	F: 5′ -AGAAGGCTGGGGCTCATTTG-3′
	Human	R: 5′ -AGGGGCCATCCACAGTCTTC-3′
PI3K	Human	F: 5′ - CGTTT CTGCT TTGGGACAAC-3′
	Human	R: 5′ - CCTGATGATGGTCGTGGAG-3′
AKT1	Human	F: 5′ - TGAGAGAAGCCACGCTGTC-3′
	Human	R: 5′ - CGGAGAACAAACTGGATGAA-3′
PCNA	Human	F: 5′ - TCTGAGGGCTTCGACACCTA-3′
	Human	R: 5′ - CATTGCCGGCGCATTTTAGT-3′
BCL2	Human	F: 5′ - GACTGAGTACCTGAACCGGC-3′
	Human	R: 5′ - TCACCAAGTGCACCTACCCA-3′
Bim	Human	F: 5′ - TGACCGAGAAGGTAGACA-3′
	Human	R: 5′ - AATAATGGCACCAGGAGA-3′

Table S2. The primary antibodies used in WB

Antibody Name	Band Size (kDa)	Diluted Multiples	Antibody Source	Company	Product Number
PCNA	36	1:1000	Rabbit	CST	13110
Caspase3	35,19,17	1:1000	Rabbit	CST	14220
Caspase8	57,18,10	1:1000	Rabbit	CST	4790
PARP	113,86	1:1000	Rabbit	Abmart	T40050
BCL2	26	1:1000	Rabbit	Abcam	ab182858
BAX	20	1:1000	Rabbit	CST	5023
p-PI3K	54,83	1:1000	Rabbit	Abmart	T40065
PI3K	54	1:1000	Rabbit	Abclonal	A18355
p-AKT1	60	1:1000	Rabbit	CST	4060
AKT1	60	1:1000	Rabbit	CST	4685
β-actin	45	1:2000	Mouse	CST	3700

Table S3. The 33 active compounds of Gou Qi Zi

ID	name	2D Structure	Database
GQZ1	Sitosterol alpha1	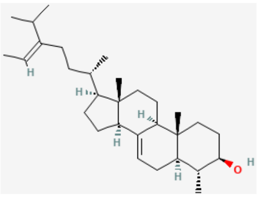	TCMSP
GQZ2	Mandenol	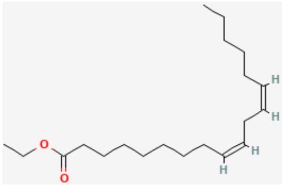	TCMSP
GQZ3	Ethyl linolenate	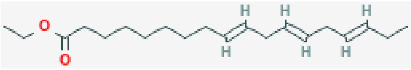	TCMSP
GQZ4	LAN	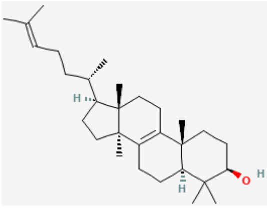	TCMSP
GQZ5	Stigmasterol	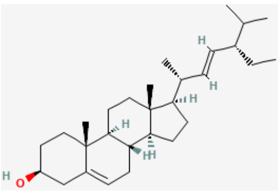	TCMSP<br>TCMID
GQZ6	beta-sitosterol	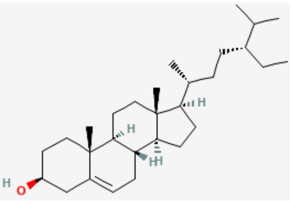	TCMSP
GQZ7	atropine	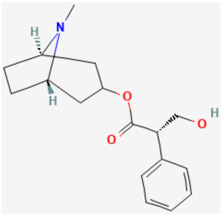	TCMSP<br>TCMID
GQZ8	campesterol	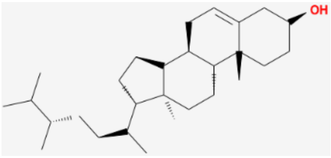	TCMSP<br>TCMID
GQZ9	cyanin	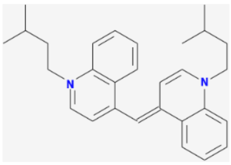	TCMSP

GQZ10	24-methylidenelophenol	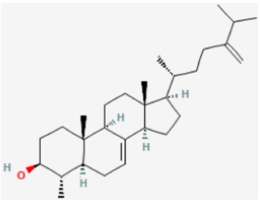	TCMSP
GQZ11	daucosterol_qt	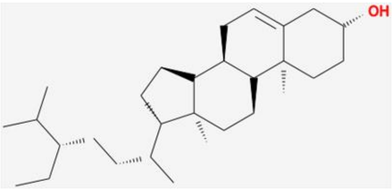	TCMSP
GQZ12	glycitein	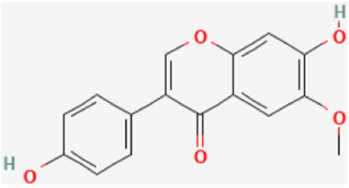	TCMSP<br>TCMID
GQZ13	CLR	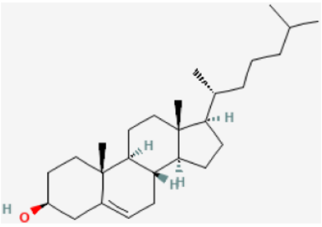	TCMSP
GQZ14	14b-pregnane	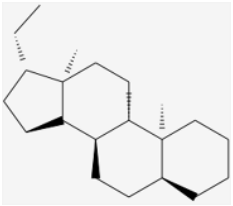	TCMSP
GQZ15	24-ethylcholest-22-enol	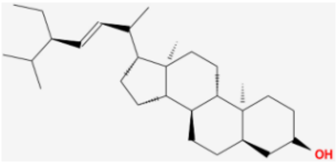	TCMSP
GQZ16	24-ethylcholesta-5,22-dienol	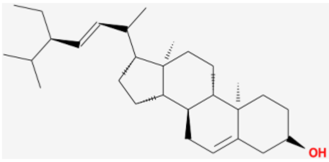	TCMSP
GQZ17	24-methyl-31-norlanost-9(11)-enol	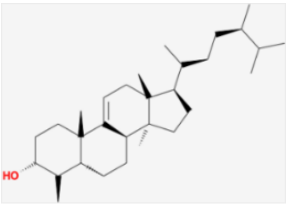	TCMSP<br>TCMID
GQZ18	24-methylenelanost-8-enol	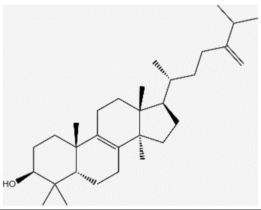	TCMSP

GQZ19	Fucosterol	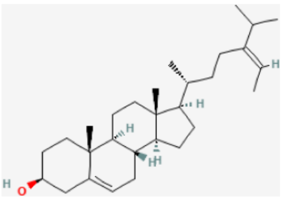	TCMSP
GQZ20	31-norlanost-9(11)-enol	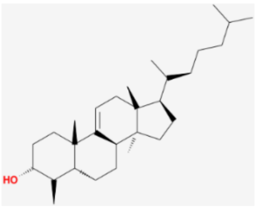	TCMSP
GQZ21	31-norlanosterol	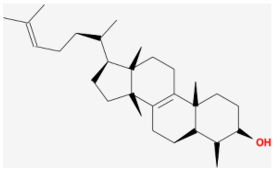	TCMSP
GQZ22	4,24-methyllophenol	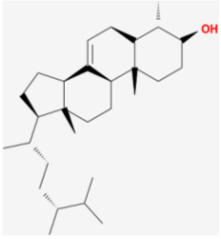	TCMSP
GQZ23	Lophenol	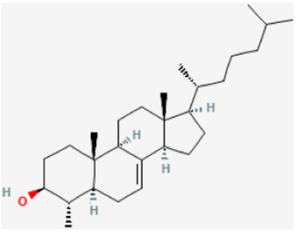	TCMSP
GQZ24	4alpha,14alpha,24-trimethylcholesta-8,24-dienol	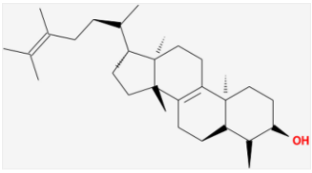	TCMSP
GQZ25	4alpha,24-dimethylcholesta-7,24-dienol	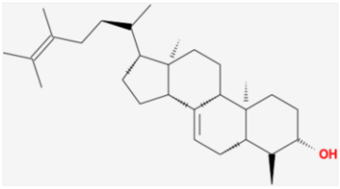	TCMSP
GQZ26	4alpha-methyl-24-ethylcholesta-7,24-dienol	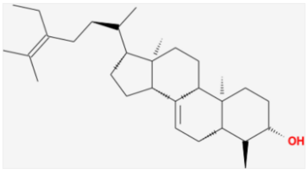	TCMSP
GQZ27	6-Fluoroindole-7-Dehydrocholesterol	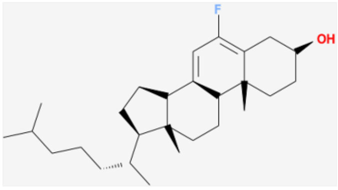	TCMSP

GQZ28	7-O-Methylluteolin-6-C-beta-glucoside_qt	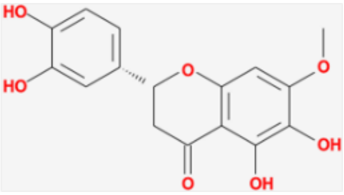	TCMSP
GQZ29	Physcion-8-O-beta-D-gentiobioside	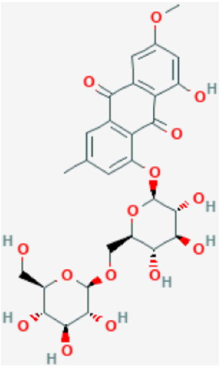	TCMSP<br>TCMID
GQZ30	lanost-8-en-3beta-ol	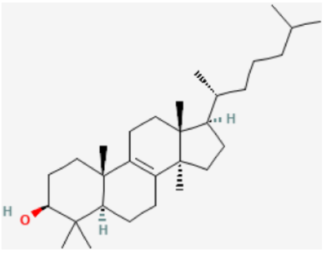	TCMSP
GQZ31	lanost-8-enol	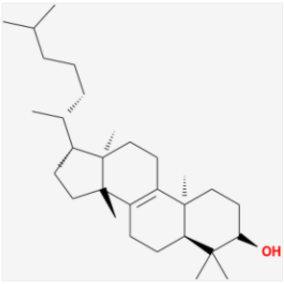	TCMSP
GQZ32	Obtusifoliol	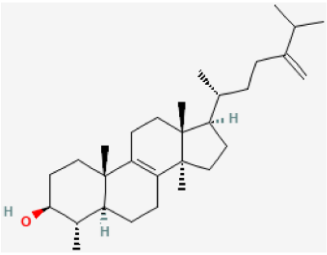	TCMSP<br>TCMID
GQZ33	quercetin	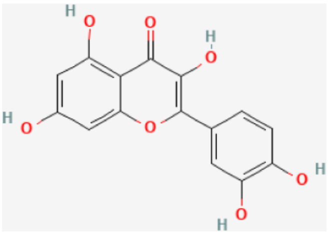	TCMSP

Table S4. Data on the Gou Qi Zi compound–predicted target network.

Name	Degree	Betweenness Centrality	Closeness Centrality	Average Shortest Path Length
GQZ1	5	0.008285803	0.352671756	2.835497835
PGR	25	0.115573441	0.312584574	3.199134199
PTGS2	9	0.051490333	0.462925852	2.16017316
GABRA1	5	0.054031414	0.453831041	2.203463203
ADH1C	2	0.000143389	0.278648975	3.588744589
NR3C2	13	0.016202694	0.286956522	3.484848485
GQZ2	3	0.000327651	0.326732673	3.060606061
PTGS1	6	0.033955256	0.457425743	2.186147186
NCOA2	15	0.09771117	0.455621302	2.194805195
GQZ3	2	0.000144190	0.32398317	3.086580087
GQZ4	3	0.002509734	0.32859175	3.043290043
GQZ5	31	0.149608383	0.384359401	2.601731602
IGHG1	1	0	0.277978339	3.597402597
RXRA	3	0.018692254	0.438330171	2.281385281
NCOA1	2	0.002992337	0.290566038	3.441558442
ADRA2A	2	0.001250964	0.287671233	3.476190476
SLC6A2	2	0.001250964	0.287671233	3.476190476
SLC6A3	2	0.001250964	0.287671233	3.476190476
ADRB2	4	0.047472807	0.452054795	2.212121212
AKR1B10	1	0	0.277978339	3.597402597
PLAU	2	0.008652787	0.417721519	2.393939394
LTA4H	1	0	0.277978339	3.597402597
MAOB	2	0.008652787	0.417721519	2.393939394
MAOA	1	0	0.277978339	3.597402597
PRKACA	4	0.027003304	0.453831041	2.203463203
CTRB1	1	0	0.277978339	3.597402597

CHRM3	3	0.00311899	0.301960784	3.311688312
CHRM1	3	0.00311899	0.301960784	3.311688312
ADRB1	2	0.001250964	0.287671233	3.476190476
SCN5A	3	0.01421244	0.433395872	2.307359307
HTR2A	3	0.00311899	0.301960784	3.311688312
ADRA1A	3	0.00311899	0.301960784	3.311688312
GABRA3	2	4.05E-04	0.293519695	3.406926407
CHRM2	3	0.00311899	0.301960784	3.311688312
ADRA1B	3	0.00311899	0.301960784	3.311688312
CHRNA7	2	0.000405329	0.293519695	3.406926407
GQZ6	37	0.17183381	0.392190153	2.54978355
HSP90AB1	5	0.021099872	0.436672968	2.29004329
PIK3CG	2	0.005154325	0.413237925	2.41991342
KCNH2	2	0.005154325	0.413237925	2.41991342
DRD5	1	0	0.282051282	3.545454545
GABRA2	1	0	0.282051282	3.545454545
CHRM4	2	0.001462697	0.292775665	3.415584416
PDE3A	2	0.002751397	0.295019157	3.38961039
GABRA5	1	0	0.282051282	3.545454545
CHRNA2	1	0	0.282051282	3.545454545
SLC6A4	2	0.001462697	0.292775665	3.415584416
OPRM1	2	0.001462697	0.292775665	3.415584416
BCL2	2	0.005154325	0.413237925	2.41991342
BAX	2	0.005154325	0.413237925	2.41991342
CASP9	2	0.005154325	0.413237925	2.41991342
JUN	2	0.005154325	0.413237925	2.41991342
CASP3	2	0.005154325	0.413237925	2.41991342
CASP8	2	0.005154325	0.413237925	2.41991342
PRKCA	2	0.005154325	0.413237925	2.41991342

TGFB1	2	0.005154325	0.413237925	2.41991342
PON1	2	0.005154325	0.413237925	2.41991342
MAP2	1	0	0.282051282	3.545454545
GQZ7	25	0.094843161	0.334298119	2.991341991
DRD1	1	0	0.250814332	3.987012987
CHRM5	1	0	0.250814332	3.987012987
ADRA2C	1	0	0.250814332	3.987012987
OPRD1	1	0	0.250814332	3.987012987
HTR2C	1	0	0.250814332	3.987012987
ADRA2B	1	0	0.250814332	3.987012987
DRD2	1	0	0.250814332	3.987012987
HTR1B	1	0	0.250814332	3.987012987
HRH1	1	0	0.250814332	3.987012987
HTR1A	1	0	0.250814332	3.987012987
GQZ8	1	0	0.238390093	4.194805195
GQZ9	2	0.000032500	0.317744154	3.147186147
GQZ10	3	0.002509734	0.32859175	3.043290043
GQZ11	2	0.001724957	0.327659574	3.051948052
GQZ12	22	0.097752754	0.337226277	2.965367965
AR	2	0.00704713	0.386934673	2.584415584
PPARG	3	0.00704713	0.386934673	2.584415584
ESR2	1	0	0.252459016	3.961038961
MAPK14	1	0	0.252459016	3.961038961
GSK3B	1	0	0.252459016	3.961038961
CDK2	1	0	0.252459016	3.961038961
CHEK1	2	0.00704713	0.386934673	2.584415584
PRSS1	2	0.00704713	0.386934673	2.584415584
PIM1	1	0	0.252459016	3.961038961
CCNA2	1	0	0.252459016	3.961038961

CALM1	2	0.000350232	0.259259259	3.857142857
NOS2	1	0	0.252459016	3.961038961
APP	1	0	0.252459016	3.961038961
MMP13	1	0	0.252459016	3.961038961
MMP8	1	0	0.252459016	3.961038961
GQZ13	3	0.002509734	0.32859175	3.043290043
GQZ14	3	0.004387127	0.340206186	2.939393939
GQZ15	1	0	0.238390093	4.194805195
GQZ16	2	0.000049527	0.238883144	4.186147186
GQZ17	1	0	0.238390093	4.194805195
GQZ18	1	0	0.238390093	4.194805195
GQZ19	3	0.002509734	0.32859175	3.043290043
GQZ20	1	0	0.238390093	4.194805195
GQZ21	3	0.002509734	0.32859175	3.043290043
GQZ22	1	0	0.238390093	4.194805195
GQZ23	1	0	0.238390093	4.194805195
GQZ24	1	0	0.238390093	4.194805195
GQZ25	3	0.002509734	0.32859175	3.043290043
GQZ26	1	0	0.238390093	4.194805195
GQZ27	3	0.008707536	0.239378238	4.177489177
NR3C1	1	0	0.193305439	5.173160173
GQZ28	5	0.004965714	0.329529244	3.034632035
TOP2A	5	0.010306385	0.373182553	2.67965368
GQZ29	1	0	0.272084806	3.675324675
GQZ30	2	0.000049527	0.238883144	4.186147186
GQZ31	2	0.000049527	0.238883144	4.186147186
GQZ32	3	0.002509734	0.32859175	3.043290043
GQZ33	152	0.806505761	0.584810127	1.70995671
DPP4	1	0	0.3696	2.705627706

AKR1B1	1	0	0.3696	2.705627706
F2R	1	0	0.3696	2.705627706
F10	1	0	0.3696	2.705627706
MMP3	1	0	0.3696	2.705627706
F7	1	0	0.3696	2.705627706
NOS3	2	0	0.3696	2.705627706
ACHE	1	0	0.3696	2.705627706
RELA	1	0	0.3696	2.705627706
EGFR	1	0	0.3696	2.705627706
AKT1	1	0	0.3696	2.705627706
VEGFA	1	0	0.3696	2.705627706
CCND1	1	0	0.3696	2.705627706
BCL2L1	1	0	0.3696	2.705627706
FOS	1	0	0.3696	2.705627706
CDKN1C	1	0	0.3696	2.705627706
EIF6	1	0	0.3696	2.705627706
MMP2	1	0	0.3696	2.705627706
MMP9	1	0	0.3696	2.705627706
MAPK1	1	0	0.3696	2.705627706
IL10	1	0	0.3696	2.705627706
EGF	1	0	0.3696	2.705627706
E2F1	2	0	0.3696	2.705627706
TNF	1	0	0.3696	2.705627706
IL6	1	0	0.3696	2.705627706
AHSA1	1	0	0.3696	2.705627706
TP53	1	0	0.3696	2.705627706
ELK1	1	0	0.3696	2.705627706
NFKBIA	1	0	0.3696	2.705627706
POR	1	0	0.3696	2.705627706

ODC1	1	0	0.3696	2.705627706
XDH	1	0	0.3696	2.705627706
TOP1	1	0	0.3696	2.705627706
RAF1	1	0	0.3696	2.705627706
SOD1	1	0	0.3696	2.705627706
MMP1	1	0	0.3696	2.705627706
HIF1A	1	0	0.3696	2.705627706
STAT1	1	0	0.3696	2.705627706
RUNX1T1	1	0	0.3696	2.705627706
CDK1	1	0	0.3696	2.705627706
HSPA5	1	0	0.3696	2.705627706
ERBB2	1	0	0.3696	2.705627706
ACACA	1	0	0.3696	2.705627706
HMOX1	1	0	0.3696	2.705627706
CYP3A4	1	0	0.3696	2.705627706
CYP1A2	1	0	0.3696	2.705627706
CAV1	1	0	0.3696	2.705627706
MYC	1	0	0.3696	2.705627706
F3	1	0	0.3696	2.705627706
GJA1	1	0	0.3696	2.705627706
CYP1A1	1	0	0.3696	2.705627706
ICAM1	1	0	0.3696	2.705627706
IL1B	1	0	0.3696	2.705627706
CCL2	1	0	0.3696	2.705627706
SELE	1	0	0.3696	2.705627706
VCAM1	1	0	0.3696	2.705627706
PTGER3	1	0	0.3696	2.705627706
CXCL8	1	0	0.3696	2.705627706
PRKCB	1	0	0.3696	2.705627706

BIRC5	1	0	0.3696	2.705627706
DUOX2	1	0	0.3696	2.705627706
HSPB1	1	0	0.3696	2.705627706
SULT1E1	1	0	0.3696	2.705627706
MGAM	1	0	0.3696	2.705627706
IL2	1	0	0.3696	2.705627706
NR1I2	1	0	0.3696	2.705627706
CYP1B1	1	0	0.3696	2.705627706
CCNB1	1	0	0.3696	2.705627706
PLAT	1	0	0.3696	2.705627706
THBD	1	0	0.3696	2.705627706
SERPINE1	1	0	0.3696	2.705627706
COL1A1	1	0	0.3696	2.705627706
IFNG	1	0	0.3696	2.705627706
ALOX5	1	0	0.3696	2.705627706
PTEN	1	0	0.3696	2.705627706
IL1A	1	0	0.3696	2.705627706
MPO	1	0	0.3696	2.705627706
NCF1	1	0	0.3696	2.705627706
ABCA2	1	0	0.3696	2.705627706
HAS2	1	0	0.3696	2.705627706
GSTP1	1	0	0.3696	2.705627706
NFE2L2	1	0	0.3696	2.705627706
NQO1	1	0	0.3696	2.705627706
TNKS	1	0	0.3696	2.705627706
AHR	1	0	0.3696	2.705627706
PSMD3	1	0	0.3696	2.705627706
SLC2A4	1	0	0.3696	2.705627706
COL3A1	1	0	0.3696	2.705627706

CXCL11	1	0	0.3696	2.705627706
CXCL2	1	0	0.3696	2.705627706
DCAF5	1	0	0.3696	2.705627706
NR1I3	1	0	0.3696	2.705627706
CHEK2	1	0	0.3696	2.705627706
INSR	1	0	0.3696	2.705627706
CLDN4	1	0	0.3696	2.705627706
PPARA	1	0	0.3696	2.705627706
PPARD	1	0	0.3696	2.705627706
HSF1	1	0	0.3696	2.705627706
CRP	1	0	0.3696	2.705627706
CXCL10	1	0	0.3696	2.705627706
CHUK	1	0	0.3696	2.705627706
SPP1	1	0	0.3696	2.705627706
RUNX2	1	0	0.3696	2.705627706
RASSF1	1	0	0.3696	2.705627706
E2F2	1	0	0.3696	2.705627706
ACP3	1	0	0.3696	2.705627706
CTSD	1	0	0.3696	2.705627706
IGFBP3	1	0	0.3696	2.705627706
IGF2	1	0	0.3696	2.705627706
CD40LG	1	0	0.3696	2.705627706
IRF4	1	0	0.3696	2.705627706
ERBB3	1	0	0.3696	2.705627706
DIO1	1	0	0.3696	2.705627706
PCOLCE	1	0	0.3696	2.705627706
NPEPPS	1	0	0.3696	2.705627706
HK2	1	0	0.3696	2.705627706
NKX3-1	1	0	0.3696	2.705627706

RASA1	1	0	0.3696	2.705627706
GSTM1	1	0	0.3696	2.705627706
GSTM2	1	0	0.3696	2.705627706

Table S5. Data on the compound–target network of Gou Qi Zi in NSCLC treatment.

Name	Degree	Betweenness Centrality	Closeness Centrality	Average Shortest Path Length
GQZ1	2	0.014543184	0.358024691	2.793103448
PGR	25	0.282698553	0.310492505	3.220689655
PTGS2	9	0.120165552	0.472312704	2.117241379
GQZ2	2	0.000163291	0.324384787	3.082758621
PTGS1	6	0.063250408	0.463258786	2.15862069
GQZ3	1	0	0.317286652	3.151724138
GQZ4	1	0	0.237315876	4.213793103
GQZ5	8	0.097916949	0.372750643	2.682758621
RXRA	3	0.028757868	0.454545455	2.2
ADRB2	4	0.036958234	0.432835821	2.310344828
AKR1B10	1	0	0.272045028	3.675862069
PLAU	2	0.012597888	0.427728614	2.337931034
PRKACA	4	0.043247215	0.457413249	2.186206897
GQZ6	13	0.159796388	0.382585752	2.613793103
PIK3CG	2	0.010379181	0.425219941	2.351724138
BCL2	2	0.010379181	0.425219941	2.351724138
BAX	2	0.010379181	0.425219941	2.351724138
CASP9	2	0.010379181	0.425219941	2.351724138
JUN	2	0.010379181	0.425219941	2.351724138
CASP3	2	0.010379181	0.425219941	2.351724138
CASP8	2	0.010379181	0.425219941	2.351724138
TGFB1	2	0.010379181	0.425219941	2.351724138

GQZ7	1	0	0.302713987	3.303448276
GQZ8	1	0	0.237315876	4.213793103
GQZ9	1	0	0.321507761	3.110344828
GQZ10	1	0	0.237315876	4.213793103
GQZ11	1	0	0.237315876	4.213793103
GQZ12	16	0.125172595	0.346062053	2.889655172
AR	2	0.012723914	0.39295393	2.544827586
PPARG	3	0.012723914	0.39295393	2.544827586
ESR2	1	0	0.257548845	3.882758621
MAPK14	1	0	0.257548845	3.882758621
GSK3B	1	0	0.257548845	3.882758621
CDK2	1	0	0.257548845	3.882758621
CHEK1	2	0.012723914	0.39295393	2.544827586
PIM1	1	0	0.257548845	3.882758621
CCNA2	1	0	0.257548845	3.882758621
NOS2	1	0	0.257548845	3.882758621
APP	1	0	0.257548845	3.882758621
MMP13	1	0	0.257548845	3.882758621
GQZ13	1	0	0.237315876	4.213793103
GQZ14	2	0.014543184	0.358024691	2.793103448
GQZ15	1	0	0.237315876	4.213793103
GQZ16	1	0	0.237315876	4.213793103
GQZ17	1	0	0.237315876	4.213793103
GQZ18	1	0	0.237315876	4.213793103
GQZ19	1	0	0.237315876	4.213793103
GQZ20	1	0	0.237315876	4.213793103
GQZ21	1	0	0.237315876	4.213793103
GQZ22	1	0	0.237315876	4.213793103
GQZ23	1	0	0.237315876	4.213793103

GQZ24	1	0	0.237315876	4.213793103
GQZ25	1	0	0.237315876	4.213793103
GQZ26	1	0	0.237315876	4.213793103
GQZ27	2	0.013793103	0.238095238	4.2
NR3C1	1	0	0.192563081	5.193103448
GQZ28	2	0.001490288	0.324384787	3.082758621
TOP2A	5	0.018322158	0.376623377	2.655172414
GQZ29	1	0	0.274102079	3.648275862
GQZ30	1	0	0.237315876	4.213793103
GQZ31	1	0	0.237315876	4.213793103
GQZ32	1	0	0.237315876	4.213793103
GQZ33	106	0.855339638	0.591836735	1.689655172
DPP4	1	0	0.372750643	2.682758621
F2R	1	0	0.372750643	2.682758621
MMP3	1	0	0.372750643	2.682758621
NOS3	2	0	0.372750643	2.682758621
RELA	1	0	0.372750643	2.682758621
EGFR	1	0	0.372750643	2.682758621
AKT1	1	0	0.372750643	2.682758621
VEGFA	1	0	0.372750643	2.682758621
CCND1	1	0	0.372750643	2.682758621
BCL2L1	1	0	0.372750643	2.682758621
FOS	1	0	0.372750643	2.682758621
CDKN1C	1	0	0.372750643	2.682758621
MMP2	1	0	0.372750643	2.682758621
MMP9	1	0	0.372750643	2.682758621
MAPK1	1	0	0.372750643	2.682758621
IL10	1	0	0.372750643	2.682758621
EGF	1	0	0.372750643	2.682758621

E2F1	2	0	0.372750643	2.682758621
TNF	1	0	0.372750643	2.682758621
IL6	1	0	0.372750643	2.682758621
TP53	1	0	0.372750643	2.682758621
NFKBIA	1	0	0.372750643	2.682758621
ODC1	1	0	0.372750643	2.682758621
TOP1	1	0	0.372750643	2.682758621
RAF1	1	0	0.372750643	2.682758621
SOD1	1	0	0.372750643	2.682758621
PRKCA	1	0	0.372750643	2.682758621
MMP1	1	0	0.372750643	2.682758621
HIF1A	1	0	0.372750643	2.682758621
STAT1	1	0	0.372750643	2.682758621
CDK1	1	0	0.372750643	2.682758621
HSPA5	1	0	0.372750643	2.682758621
ERBB2	1	0	0.372750643	2.682758621
HMOX1	1	0	0.372750643	2.682758621
CYP3A4	1	0	0.372750643	2.682758621
CYP1A2	1	0	0.372750643	2.682758621
CAV1	1	0	0.372750643	2.682758621
MYC	1	0	0.372750643	2.682758621
F3	1	0	0.372750643	2.682758621
CYP1A1	1	0	0.372750643	2.682758621
ICAM1	1	0	0.372750643	2.682758621
IL1B	1	0	0.372750643	2.682758621
CCL2	1	0	0.372750643	2.682758621
SELE	1	0	0.372750643	2.682758621
CXCL8	1	0	0.372750643	2.682758621
BIRC5	1	0	0.372750643	2.682758621

HSPB1	1	0	0.372750643	2.682758621
IL2	1	0	0.372750643	2.682758621
CYP1B1	1	0	0.372750643	2.682758621
CCNB1	1	0	0.372750643	2.682758621
SERPINE1	1	0	0.372750643	2.682758621
COL1A1	1	0	0.372750643	2.682758621
IFNG	1	0	0.372750643	2.682758621
ALOX5	1	0	0.372750643	2.682758621
PTEN	1	0	0.372750643	2.682758621
IL1A	1	0	0.372750643	2.682758621
MPO	1	0	0.372750643	2.682758621
GSTP1	1	0	0.372750643	2.682758621
NFE2L2	1	0	0.372750643	2.682758621
NQO1	1	0	0.372750643	2.682758621
AHR	1	0	0.372750643	2.682758621
CHEK2	1	0	0.372750643	2.682758621
INSR	1	0	0.372750643	2.682758621
CLDN4	1	0	0.372750643	2.682758621
PPARA	1	0	0.372750643	2.682758621
HSF1	1	0	0.372750643	2.682758621
CRP	1	0	0.372750643	2.682758621
CXCL10	1	0	0.372750643	2.682758621
CHUK	1	0	0.372750643	2.682758621
SPP1	1	0	0.372750643	2.682758621
RASSF1	1	0	0.372750643	2.682758621
CTSD	1	0	0.372750643	2.682758621
IGFBP3	1	0	0.372750643	2.682758621
IGF2	1	0	0.372750643	2.682758621
CD40LG	1	0	0.372750643	2.682758621

IRF4	1	0	0.372750643	2.682758621
ERBB3	1	0	0.372750643	2.682758621
HK2	1	0	0.372750643	2.682758621
RASA1	1	0	0.372750643	2.682758621
GSTM1	1	0	0.372750643	2.682758621
GJA1	1	0	0.372750643	2.682758621
THBD	1	0	0.372750643	2.682758621
RUNX2	1	0	0.372750643	2.682758621

Table S6. Data on reconstructed PPI network.

Name	Degree	Betweenness Centrality	Closeness Centrality	Average Shortest Path Length
AKT1	100	0.052834537	0.903225806	1.107142857
TP53	98	0.048673064	0.888888889	1.125
VEGFA	91	0.030333115	0.842105263	1.1875
JUN	87	0.02464945	0.817518248	1.223214286
CASP3	86	0.024463592	0.811594203	1.232142857
TNF	85	0.023338202	0.805755396	1.241071429
MYC	84	0.030656954	0.8	1.25
EGFR	83	0.038687242	0.794326241	1.258928571
IL6	83	0.019841457	0.794326241	1.258928571
HIF1A	82	0.021726153	0.788732394	1.267857143
IL1B	79	0.015525783	0.772413793	1.294642857
PTGS2	77	0.014744455	0.761904762	1.3125
EGF	76	0.014138621	0.751677852	1.330357143
CCND1	75	0.018502853	0.751677852	1.330357143
MMP9	75	0.011537685	0.746666667	1.339285714
PTEN	72	0.01710745	0.736842105	1.357142857
PPARG	71	0.014387655	0.732026144	1.366071429

FOS	68	0.011103507	0.717948718	1.392857143
CXCL8	65	0.007750135	0.7	1.428571429
ERBB2	64	0.018985312	0.695652174	1.4375
CCL2	63	0.006167692	0.691358025	1.446428571
IL10	61	0.005351299	0.682926829	1.464285714
CASP8	61	0.008697403	0.682926829	1.464285714
NFKBIA	60	0.00593526	0.682926829	1.464285714
MMP2	60	0.00530255	0.674698795	1.482142857
HMOX1	59	0.00791736	0.674698795	1.482142857
BCL2L1	58	0.006497258	0.670658683	1.491071429
MAPK1	57	0.009971966	0.666666667	1.5
RELA	55	0.004492142	0.658823529	1.517857143
ICAM1	54	0.00335323	0.65497076	1.526785714
IFNG	52	0.002268892	0.647398844	1.544642857
NOS3	51	0.004740525	0.643678161	1.553571429
TGFB1	51	0.001834786	0.643678161	1.553571429
CAV1	49	0.004442816	0.636363636	1.571428571
STAT1	49	0.002786137	0.636363636	1.571428571
CASP9	49	0.003303074	0.632768362	1.580357143
IL2	48	0.001791286	0.632768362	1.580357143
PPARA	48	0.011048502	0.636363636	1.571428571
SERPINE1	48	0.002724713	0.632768362	1.580357143
MAPK14	48	0.002415366	0.632768362	1.580357143
AR	47	0.005190749	0.629213483	1.589285714
GSK3B	45	0.002986064	0.622222222	1.607142857
PGR	43	0.005469721	0.615384615	1.625
SPP1	43	0.002138921	0.615384615	1.625
NR3C1	42	0.003071684	0.612021858	1.633928571
IL1A	42	0.001244508	0.612021858	1.633928571

CRP	41	0.001776137	0.608695652	1.642857143
MMP3	41	0.001096372	0.605405405	1.651785714
MMP1	40	0.001034319	0.602150538	1.660714286
NFE2L2	39	0.002163041	0.605405405	1.651785714
MPO	39	0.002627419	0.602150538	1.660714286
CDK2	38	0.002323149	0.592592593	1.6875
HSPA5	38	0.002166152	0.602150538	1.660714286
NOS2	38	0.002160246	0.595744681	1.678571429
CXCL10	37	0.000866	0.586387435	1.705357143
AHR	36	0.005750842	0.595744681	1.678571429
HSPB1	36	0.001487928	0.589473684	1.696428571
CCNB1	36	0.001674326	0.589473684	1.696428571
APP	36	0.001109524	0.589473684	1.696428571
SELE	36	0.000577	0.589473684	1.696428571
RUNX2	36	0.001427257	0.589473684	1.696428571
CD40LG	35	0.001541348	0.580310881	1.723214286
IGFBP3	35	0.00151796	0.586387435	1.705357143
PRKCA	35	0.001890096	0.589473684	1.696428571
PLAU	34	0.001318922	0.589473684	1.696428571
CDK1	33	0.00276815	0.580310881	1.723214286
IGF2	33	0.002107811	0.580310881	1.723214286
ESR2	32	0.001841793	0.580310881	1.723214286
RAF1	32	0.001857209	0.577319588	1.732142857
CHUK	31	0.000213	0.574358974	1.741071429
CCNA2	30	0.000826	0.571428571	1.75
NQO1	29	0.015085145	0.571428571	1.75
MMP13	29	0.000276	0.565656566	1.767857143
F3	29	0.000736	0.568527919	1.758928571
COL1A1	28	0.000274	0.565656566	1.767857143

SOD1	27	0.001357597	0.565656566	1.767857143
BAX	27	0.000951	0.56281407	1.776785714
GJA1	26	0.000329	0.56	1.785714286
CTSD	25	0.000657	0.55721393	1.794642857
CHEK1	25	0.000541	0.55721393	1.794642857
GSTP1	24	0.003342495	0.56	1.785714286
E2F1	23	0.00076	0.551724138	1.8125
CYP1A1	23	0.002401903	0.549019608	1.821428571
BIRC5	23	0.001116755	0.549019608	1.821428571
DPP4	23	0.000393	0.549019608	1.821428571
ERBB3	22	0.000542	0.549019608	1.821428571
TOP1	22	0.000251	0.549019608	1.821428571
CHEK2	22	0.000399	0.549019608	1.821428571
IRF4	21	0.000145	0.546341463	1.830357143
PRKACA	21	0.001048428	0.546341463	1.830357143
F2R	20	0.000817	0.538461538	1.857142857
CYP3A4	20	0.002371972	0.549019608	1.821428571
TOP2A	19	0.000291	0.535885167	1.866071429
THBD	19	0.000157	0.513761468	1.946428571
ALOX5	18	0.0000837	0.535885167	1.866071429
BCL2	18	0.000548	0.533333333	1.875
INSR	17	0.000883	0.535885167	1.866071429
PIK3CG	17	0.000707	0.535885167	1.866071429
PTGS1	17	0.000148	0.528301887	1.892857143
RXRA	17	0.000956	0.535885167	1.866071429
CYP1B1	16	0.00160184	0.533333333	1.875
GSTM1	14	0.000931	0.523364486	1.910714286
RASA1	14	0.000283	0.520930233	1.919642857
ADRB2	13	0.000599	0.528301887	1.892857143

HK2	13	0.006581908	0.530805687	1.883928571
CDKN1C	12	0.0000518	0.518518519	1.928571429
ODC1	11	0.0000711	0.520930233	1.919642857
RASSF1	11	0.0000506	0.520930233	1.919642857
HSF1	11	0.000168	0.516129032	1.9375
CYP1A2	8	0.0000576	0.4375	2.285714286
PIM1	7	0.0000572	0.49122807	2.035714286
AKR1B10	2	0.000023	0.375838926	2.660714286
CLDN4	2	0	0.449799197	2.223214286

Table S7. Annotation of biological processes.

Term ID	Description	*P* value	Gene count	Gene ratio (%)
GO:0032496	response to lipopolysaccharide	1.22E-40	35	30.9735
GO:0010035	response to inorganic substance	5.94E-40	40	35.3982
GO:0009611	response to wounding	1.16E-37	38	33.6283
GO:0071407	cellular response to organic cyclic compound	1.12E-36	38	33.6283
GO:0097190	apoptotic signaling pathway	2.35E-35	37	32.7434
GO:0001568	blood vessel development	1.14E-33	39	34.5133
GO:0009410	response to xenobiotic stimulus	1.24E-31	32	28.3186
GO:0030155	regulation of cell adhesion	9.45E-29	35	30.9735
GO:0008285	negative regulation of cell population proliferation	3.26E-28	35	30.9735
GO:1901652	response to peptide	1.43E-27	30	26.5487
GO:0070848	response to growth factor	8.58E-27	33	29.2035
GO:0070482	response to oxygen levels	1.48E-26	26	23.0088
GO:0050673	epithelial cell proliferation	2.12E-26	28	24.7788
GO:0010942	positive regulation of cell death	2.37E-26	31	27.4336
GO:0009991	response to extracellular stimulus	2.69E-26	29	25.6637
GO:0018105	peptidyl-serine phosphorylation	3.61E-26	25	22.1239

GO:0009314	response to radiation	3.66E-26	28	24.7788
GO:0070997	neuron death	4.14E-26	26	23.0088
GO:0072593	reactive oxygen species metabolic process	4.71E-26	23	20.3540
GO:0033002	muscle cell proliferation	1.12E-25	23	20.3540

Table S8. Annotation of molecular function.

Term ID	Description	*P* value	Gene count	Gene ratio (%)
GO:0019904	protein domain specific binding	8.34E-19	26	23.0088
GO:0008134	transcription factor binding	1.77E-18	24	21.2389
GO:0019901	protein kinase binding	4.63E-17	24	21.2389
GO:0004672	protein kinase activity	3.87E-15	21	18.5841
GO:0005126	cytokine receptor binding	5.65E-15	16	14.1593
GO:0044389	ubiquitin-like protein ligase binding	9.99E-13	15	13.2743
GO:0042803	protein homodimerization activity	8.90E-12	19	16.8142
GO:0004879	nuclear receptor activity	1.94E-11	8	7.07965
GO:0019207	kinase regulator activity	8.20E-11	12	10.6195
GO:0002020	protease binding	9.42E-11	10	8.8496
GO:0020037	heme binding	1.26E-10	10	8.8496
GO:0019902	phosphatase binding	3.61E-09	10	8.84956
GO:0046982	protein heterodimerization activity	4.03E-09	12	10.6195
GO:0004175	endopeptidase activity	9.93E-08	12	10.6195
GO:0035173	histone kinase activity	3.25E-07	3	3.5398
GO:0031072	heat shock protein binding	5.79E-07	7	6.1947
GO:0016209	antioxidant activity	9.40E-07	6	5.30973
GO:0032813	tumor necrosis factor receptor superfamily binding	1.11E-06	5	4.4248
GO:0005178	integrin binding	1.21E-06	7	6.1947
GO:0097199	cysteine-type endopeptidase activity involved in apoptotic signaling pathway	5.98E-06	3	2.6549

Table S9. Annotation of cellular component.

Term ID	Description	*P* value	Gene count	Gene ratio (%)
GO:0045121	membrane raft	5.77E-16	18	15.9292
GO:0031983	vesicle lumen	3.17E-10	13	11.5044
GO:0031968	organelle outer membrane	1.67E-08	10	8.8496
GO:1902911	protein kinase complex	7.64E-08	7	6.1947
GO:0005819	spindle	2.62E-07	11	9.7345
GO:0005667	transcription regulator complex	4.51E-07	11	9.7345
GO:0048471	perinuclear region of cytoplasm	6.94E-07	14	12.3894
GO:0031012	extracellular matrix	1.57E-06	12	10.6195
GO:0005813	centrosome	3.43E-06	12	10.6195
GO:0043235	receptor complex	4.93E-06	11	9.7345
GO:1904813	ficolin-1-rich granule lumen	7.43E-06	6	5.3097
GO:0005788	endoplasmic reticulum lumen	2.40E-05	8	7.0796
GO:0098552	side of membrane	2.81E-05	11	9.7345
GO:0005635	nuclear envelope	6.79E-05	9	7.9646
GO:0000323	lytic vacuole	9.28E-05	11	9.7345
GO:0045177	apical part of cell	0.000243178	8	7.0796
GO:0030139	endocytic vesicle	0.000277064	7	6.1947
GO:0098687	chromosomal region	0.00034224	7	6.1947
GO:0120111	neuron projection cytoplasm	0.00041768	4	3.5398
GO:0016605	PML body	0.000637399	3	3.5398

Table S10. Annotation of KEGG pathway

Term ID	Description	Count	*P* value	Genes
hsa05200	Pathways in cancer	53	1.34E-60	AKT1,BIRC5,AR,BAX,CCND1,BCL2, BCL2L1,CASP3,CASP8,CASP9,CDK2, CHUK,NQO1,E2F1,EGF,EGFR,ERBB2, ESR2,F2R,FOS,GSK3B,GSTM1,GSTP1, HIF1A,HMOX1,IFNG,IGF2,IL2,IL6,

				CXCL8,JUN,MMP1,MMP2,MMP9,MYC, NFE2L2,NFKBIA,NOS2,PIM1,PPARG, PRKACA,PRKCA,MAPK1,PTEN,PTGS2, RAF1,RELA,RXRA,STAT1,TGFB1,T P53,VEGFA,RASSF1
hsa04933	AGE-RAGE signaling pathway in diabetic complications	28	1.02E-44	AKT1,BAX,CCND1,BCL2,CASP3, COL1A1,MAPK14,F3,ICAM1,IL1A, IL1B,IL6,CXCL8,JUN,MMP2,NOS3, SERPINE1,PIM1,PRKCA,MAPK1, RELA,CCL2,SELE,STAT1,TGFB1, THBD,TNF,VEGFA
hsa05161	Hepatitis B	29	8.19E-40	AKT1,BIRC5,BAX,CCND1,BCL2, CASP3,CASP8,CASP9,CCNA2,CDK2, CHUK,MAPK14,E2F1,FOS,IL6,CXCL8, JUN,MMP9,MYC,NFKBIA,PRKCA, MAPK1,PTEN,RAF1,RELA,STAT1, TGFB1,TNF,TP53
hsa05160	Hepatitis C	27	9.68E-37	AKT1,BAX,CCND1,CASP3,CASP8, CASP9,CDK2,CHUK,CLDN4,MAPK14 ,E2F1,EGF,EGFR,GSK3B,IFNG,CXCL8, CXCL10,MYC,NFKBIA,PPARA,MAPK1, RAF1,RELA,RXRA,STAT1,TNF,TP53
hsa05418	Fluid shear stress and atherosclerosis	26	1.56E-36	AKT1,BCL2,CAV1,CHUK,MAPK14, NQO1,FOS,GSTM1,GSTP1,HMOX1, ICAM1,IFNG,IL1A,IL1B,JUN,MMP2, MMP9,NFE2L2,NOS3,RELA,CCL2, SELE,THBD,TNF,TP53,VEGFA
hsa04657	IL-17signaling pathway	22	7.39E-34	CASP3,CASP8,CHUK,MAPK14, FOS,GSK3B,IFNG,IL1B,IL6,CXCL8, CXCL10,JUN,MMP1,MMP3, MMP9,MMP13,NFKBIA,MAPK1, PTGS2,RELA,CCL2,TNF
hsa05215	Prostate cancer	22	4.21E-33	AKT1,AR,CCND1,BCL2,CASP9, CDK2,CHUK,E2F1,EGF,EGFR, ERBB2,GSK3B,GSTP1,MMP3, MMP9,NFKBIA,PLAU,MAPK1, PTEN,RAF1,RELA,TP53
hsa04151	PI3K-Akt signaling pathway	30	2.47E-31	AKT1,CCND1,BCL2,BCL2L1, CASP9,CDK2,CHUK,COL1A1, EGF,EGFR,ERBB2,ERBB3,F2R, GSK3B,IGF2,IL2,IL6,INSR,MYC, NOS3,PIK3CG,PRKCA,MAPK1, PTEN,RAF1,RELA,RXRA,SPP1, TP53,VEGFA

hsa05169	Epstein-Barr virus infection	28	2.48E-31	AKT1,BAX,CCND1,BCL2,CASP3, CASP8,CASP9,CCNA2,CDK1, CDK2,CHUK,MAPK14,E2F1, GSK3B,HSPB1,ICAM1,IFNG,IL6, IL10,CXCL10,JUN,MYC,NFKBIA, PRKACA,RELA,STAT1,TNF,TP53
hsa04010	MAPK signaling pathway	27	5.67E-29	AKT1,CASP3,CHUK,MAPK14, EGF,EGFR,ERBB2,ERBB3,FOS, HSPB1,IGF2,IL1A,IL1B,IL6,INSR, JUN,MYC,PRKACA,PRKCA, MAPK1,RAF1,RASA1,RELA, TGFB1,TNF,TP53,VEGFA
hsa05142	Chagas disease (American trypanosomiasis)	20	8.78E-29	AKT1,CASP8,CHUK,MAPK14, FOS,IFNG,IL1B,IL2,IL6,CXCL8, IL10,JUN,NFKBIA,NOS2,SERPINE1, MAPK1,RELA,CCL2,TGFB1,TNF
hsa05162	Measles	22	1.35E-28	AKT1,BAX,CCND1,BCL2, BCL2L1,CASP3,CASP8,CASP9, CDK2,CHUK,FOS,GSK3B,IFNG, IL1A,IL1B,IL2,IL6,JUN,NFKBIA, RELA,STAT1,TP53
hsa04668	TNF signaling pathway	20	4.17E-28	AKT1,CASP3,CASP8,CHUK, MAPK14,FOS,ICAM1,IL1B,IL6, CXCL10,JUN,MMP3,MMP9, NFKBIA,MAPK1,PTGS2, RELA,CCL2,SELE,TNF
hsa05145	Toxoplasmosis	20	5.03E-28	AKT1,ALOX5,BCL2,BCL2L1, CASP3,CASP8,CASP9,CD40LG, CHUK,MAPK14,IFNG,IL10, NFKBIA,NOS2,PIK3CG,MAPK1, RELA,STAT1,TGFB1,TNF
hsa05205	Proteoglycans in cancer	23	5.41E-27	AKT1,CCND1,CASP3,CAV1, MAPK14,EGFR,ERBB2,ERBB3, HIF1A,IGF2,IL6,MMP2,MMP9, MYC,PLAU,PRKACA,PRKCA, MAPK1,RAF1,TGFB1,TNF, TP53,VEGFA
hsa05219	Bladder cancer	15	3.10E-26	CCND1,E2F1,EGF,EGFR, ERBB2,CXCL8,MMP1,MMP2, MMP9,MYC,MAPK1,RAF1, TP53,VEGFA,RASSF1
hsa04066	HIF-1 signaling pathway	19	5.57E-26	AKT1,BCL2,EGF,EGFR,ERBB2, F3,HIF1A,HK2,HMOX1,IFNG, IL6,INSR,NOS2,NOS3,SERPINE1,

				PRKCA,MAPK1,RELA,VEGFA
hsa05222	Small cell lung cancer	18	9.57E-26	AKT1,BAX,CCND1,BCL2, BCL2L1,CASP3,CASP9,CDK2, CHUK,E2F1,MYC,NFKBIA,NOS2, PTEN,PTGS2,RELA,RXRA,TP53
hsa05212	Pancreatic cancer	17	2.03E-25	AKT1,BAX,CCND1,BCL2L1, CASP9,CHUK,E2F1,EGF,EGFR, ERBB2,MAPK1,RAF1,RELA, STAT1,TGFB1,TP53,VEGFA
hsa05210	Colorectal cancer	17	2.55E-24	AKT1,BIRC5,BAX,CCND1, BCL2,CASP3,CASP9,EGF, EGFR,FOS,GSK3B,JUN,MYC, MAPK1,RAF1,TGFB1,TP53
hsa01522	Endocrine resistance	17	3.75E-24	AKT1,BAX,CCND1,BCL2, MAPK14,E2F1,EGFR,ERBB2, ESR2,FOS,JUN,MMP2,MMP9, PRKACA,MAPK1,RAF1,TP53
hsa05164	Influenza A	20	6.51E-24	AKT1,CASP9,MAPK14,GSK3B, ICAM1,IFNG,IL1A,IL1B,IL6, CXCL8,CXCL10,JUN,NFKBIA, PRKCA,MAPK1,RAF1,RELA, CCL2,STAT1,TNF
hsa04115	p53 signaling pathway	16	8.48E-24	BAX,CCND1,BCL2,BCL2L1, CASP3,CASP8,CASP9,CCNB1, CDK1,CDK2,CHEK1,IGFBP3, SERPINE1,PTEN,TP53,CHEK2
hsa05152	Tuberculosis	20	1.95E-23	AKT1,BAX,BCL2,CASP3,CASP8, CASP9,MAPK14,CTSD,IFNG, IL1A,IL1B,IL6,IL10,NOS2,MAPK1, RAF1,RELA,STAT1,TGFB1,TNF

Table S11. Data on the target–pathway network in KEGG enrichment.

Name	Degree	Betweenness Centrality	Closeness Centrality	Average Shortest Path Length
hsa05200	53	0.23167864	0.5410628	1.84821429
hsa04151	30	0.08104929	0.44268775	2.25892857
hsa05161	29	0.0374263	0.43921569	2.27678571
hsa05169	28	0.05763222	0.43579767	2.29464286
hsa04933	28	0.06864227	0.43579767	2.29464286
hsa04010	27	0.05826682	0.43243243	2.3125

hsa05160	27	0.05844889	0.43243243	2.3125
hsa05418	26	0.05908866	0.42911877	2.33035714
hsa05205	23	0.03839083	0.41947566	2.38392857
hsa05162	22	0.01759878	0.41635688	2.40178571
hsa05215	22	0.03359069	0.41635688	2.40178571
hsa04657	22	0.04075704	0.41635688	2.40178571
AKT1	21	0.03659652	0.51851852	1.92857143
hsa05152	20	0.03182363	0.41025641	2.4375
hsa05164	20	0.01636848	0.41025641	2.4375
hsa05145	20	0.05313228	0.41025641	2.4375
hsa04668	20	0.02323937	0.41025641	2.4375
hsa05142	20	0.02017047	0.41025641	2.4375
hsa04066	19	0.04350682	0.40727273	2.45535714
RELA	19	0.03268755	0.51376147	1.94642857
MAPK1	19	0.0322501	0.50909091	1.96428571
hsa05222	18	0.01285583	0.40433213	2.47321429
hsa01522	17	0.01264109	0.40143369	2.49107143
hsa05210	17	0.00950686	0.40143369	2.49107143
hsa05212	17	0.00859509	0.40143369	2.49107143
hsa04115	16	0.08015036	0.39857651	2.50892857
TP53	16	0.02676484	0.49122807	2.03571429
hsa05219	15	0.01065164	0.39575972	2.52678571
CHUK	15	0.0191918	0.48275862	2.07142857
CASP3	15	0.02767176	0.5045045	1.98214286
BCL2	15	0.02908262	0.49557522	2.01785714
CCND1	15	0.0223282	0.48695652	2.05357143
MAPK14	14	0.01538427	0.46666667	2.14285714
IL6	14	0.01754702	0.47457627	2.10714286
CASP9	14	0.02008338	0.47863248	2.08928571

TNF	13	0.01355224	0.45901639	2.17857143
RAF1	13	0.01135617	0.4516129	2.21428571
JUN	13	0.01169326	0.4516129	2.21428571
NFKBIA	12	0.00989489	0.44094488	2.26785714
BAX	12	0.01381314	0.4516129	2.21428571
IFNG	11	0.01206254	0.45901639	2.17857143
EGFR	11	0.00922584	0.4375	2.28571429
CASP8	11	0.01558385	0.4516129	2.21428571
TGFB1	10	0.00804664	0.44444444	2.25
STAT1	10	0.00750243	0.44094488	2.26785714
MYC	10	0.00734295	0.43410853	2.30357143
FOS	10	0.00667985	0.43076923	2.32142857
IL1B	9	0.00482599	0.4028777	2.48214286
VEGFA	9	0.00816119	0.43076923	2.32142857
MMP9	9	0.00618047	0.41481481	2.41071429
GSK3B	9	0.0062912	0.43410853	2.30357143
ERBB2	9	0.00626845	0.42105263	2.375
EGF	9	0.00667557	0.42748092	2.33928571
E2F1	9	0.00440616	0.4028777	2.48214286
CDK2	9	0.00951426	0.44444444	2.25
PRKCA	8	0.00620537	0.43410853	2.30357143
CXCL8	8	0.00513936	0.42105263	2.375
BCL2L1	7	0.00691947	0.41791045	2.39285714
CCL2	6	0.00182497	0.36129032	2.76785714
IL1A	6	0.0021432	0.38356164	2.60714286
PTEN	6	0.00531029	0.40875912	2.44642857
NOS2	6	0.00370471	0.4028777	2.48214286
MMP2	6	0.00302362	0.4028777	2.48214286
CXCL10	5	0.00116209	0.34782609	2.875

ICAM1	5	0.00142828	0.36601307	2.73214286
PRKACA	5	0.00198933	0.4	2.5
IL10	4	0.000782	0.33939394	2.94642857
SERPINE1	4	0.00384596	0.35897436	2.78571429
NOS3	4	0.00170113	0.37583893	2.66071429
RXRA	4	0.00131982	0.3862069	2.58928571
PTGS2	4	0.00122385	0.38095238	2.625
IL2	4	0.00118118	0.39160839	2.55357143
IGF2	4	0.00145005	0.3943662	2.53571429
INSR	3	0.000699	0.34567901	2.89285714
ERBB3	3	0.000474	0.34146341	2.92857143
MMP3	3	0.000387	0.32748538	3.05357143
SELE	3	0.000413	0.3373494	2.96428571
MMP1	3	0.000806	0.37333333	2.67857143
HMOX1	3	0.0011668	0.39160839	2.55357143
HIF1A	3	0.000984	0.38095238	2.625
GSTP1	3	0.0008	0.3862069	2.58928571
BIRC5	3	0.000327	0.36363636	2.75
HSPB1	2	0.000191	0.33333333	3
CDK1	2	0.000533	0.31818182	3.14285714
PIK3CG	2	0.000398	0.33136095	3.01785714
PLAU	2	0.000119	0.32	3.125
CAV1	4	0.000202	0.32941176	3.03571429
CCNA2	4	0.0000615	0.32183908	3.10714286
THBD	4	0.000137	0.3255814	3.07142857
F3	4	0.000225	0.32183908	3.10714286
COL1A1	4	0.000327	0.34355828	2.91071429
RASSF1	4	0.000176	0.35443038	2.82142857
PIM1	4	0.000438	0.38356164	2.60714286

NFE2L2	4	0.000347	0.37837838	2.64285714
GSTM1	4	0.000347	0.37837838	2.64285714
F2R	4	0.000351	0.36842105	2.71428571
ESR2	4	0.00015	0.3566879	2.80357143
NQO1	4	0.000347	0.37837838	2.64285714
AR	4	0.000203	0.35897436	2.78571429
CTSD	4	0	0.29166667	3.42857143
CHEK2	4	0	0.28571429	3.5
IGFBP3	4	0	0.28571429	3.5
CHEK1	4	0	0.28571429	3.5
CCNB1	4	0	0.28571429	3.5
HK2	4	0	0.29015544	3.44642857
CD40LG	4	0	0.29166667	3.42857143
ALOX5	4	0	0.29166667	3.42857143
RASA1	1	0	0.3027027	3.30357143
SPP1	1	0	0.30769231	3.25
MMP13	1	0	0.29473684	3.39285714
PPARA	1	0	0.3027027	3.30357143
CLDN4	1	0	0.3027027	3.30357143
PPARG	1	0	0.35220126	2.83928571

2. Supplementary Figures

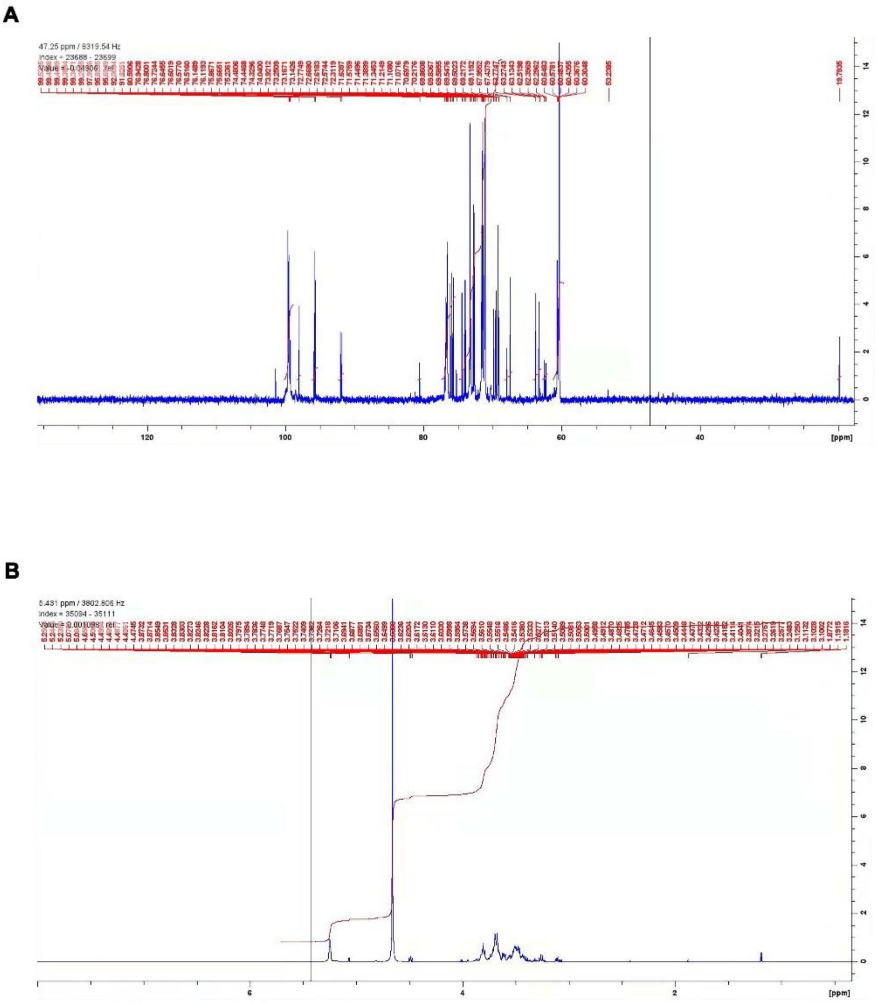

Figure S1. Method of obtaining the *Lycium barbarum* (LB) extract and assays of its purity.

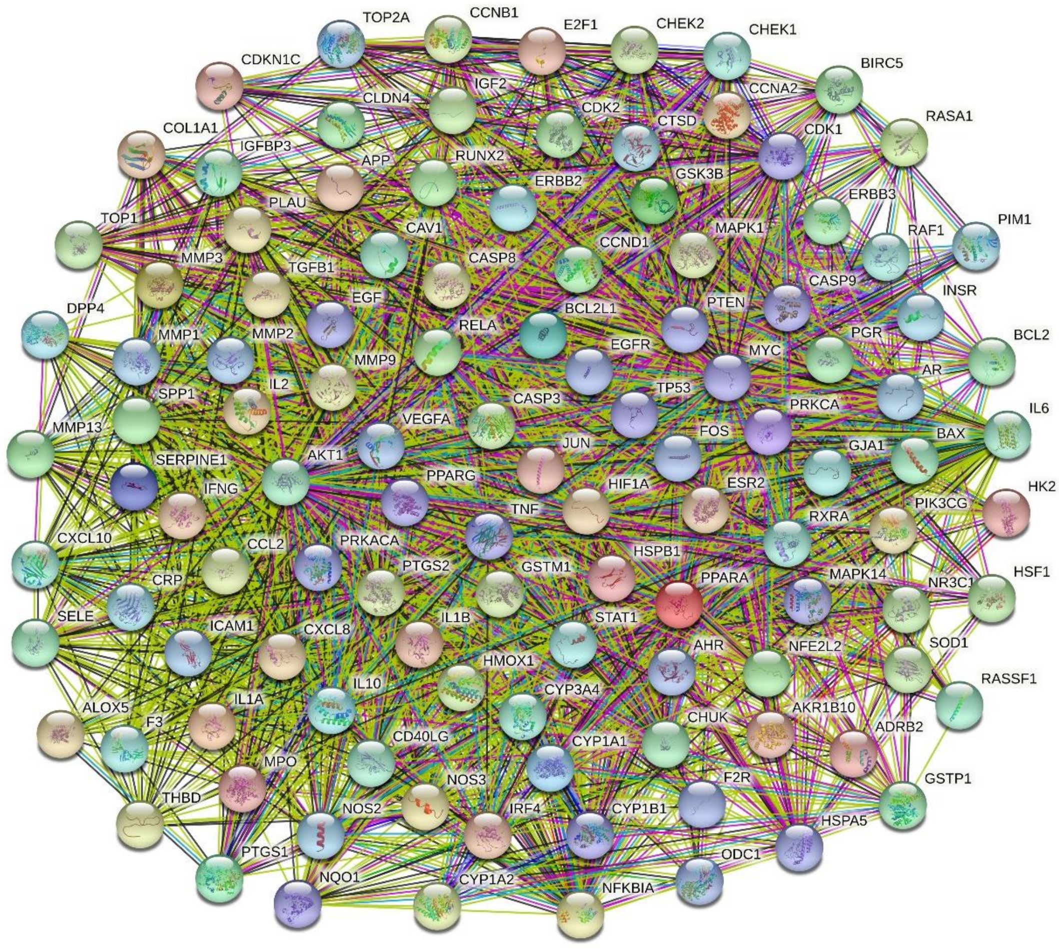

Figure S2. Original PPI network of the NSCLC targets of GQZ obtained from the STRING database.

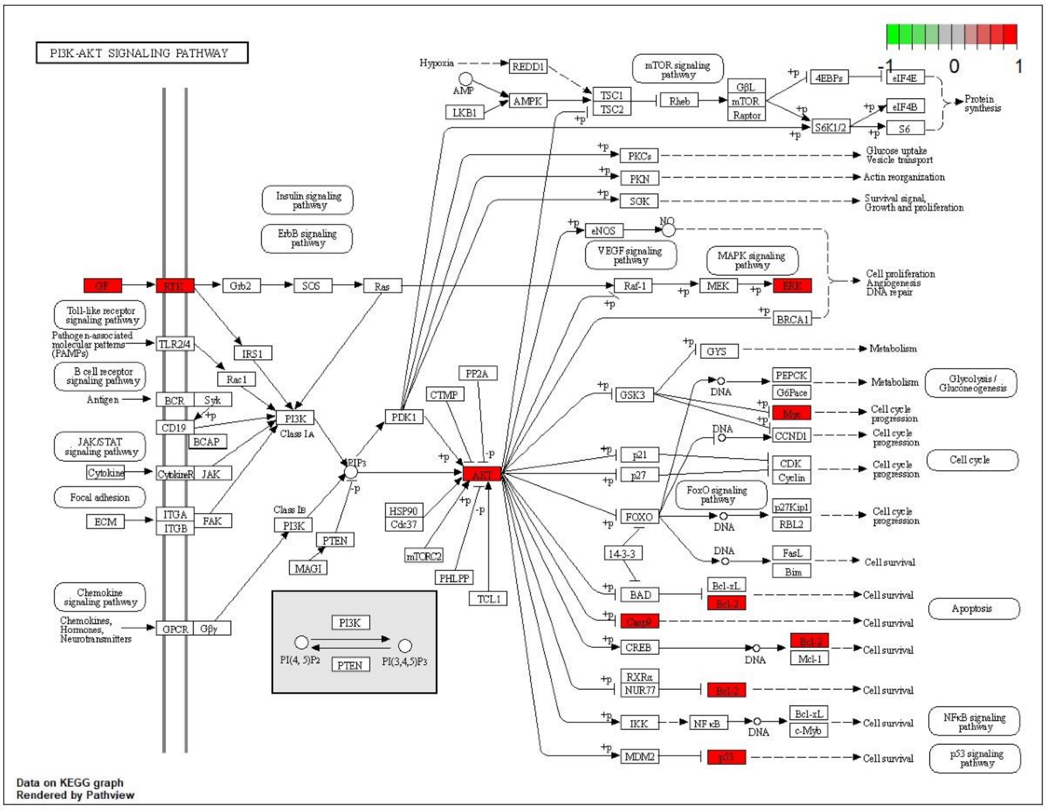

Figure S3. PI3K/AKT signaling pathway adapted from the KEGG (ID: hsa04151). The targets in this pathway are shown in red.

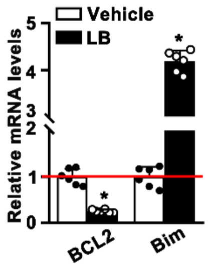

Figure S4. Effects of LB on expression of BCL2 and Bim mRNAs. A549 cells were treated with LB (80 μg/ml) or vehicle for 24 h, and the levels of BCL2 and Bim mRNAs were analyzed by qRT-PCR, with GAPDH mRNA used as the internal control.

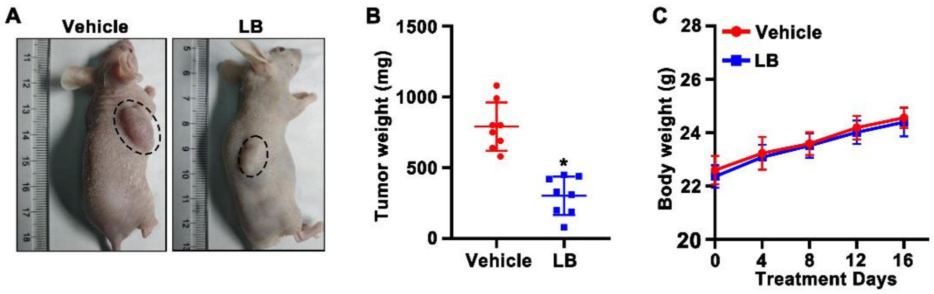

Figure S5. Effects of LB (10 mg/kg/day) on A549 xenograft tumor growth and mouse body weights. (A) Representative pictures of tumors in vivo. (B) Weights of xenograft tumors after 16 days of LB injection. (C) Body weight-time curve in mice.

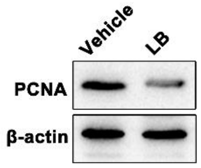

Figure S6. Effects of LB on the expression level of PCNA protein in A549 xenograft tumors, as determined by western blotting.
